# Ionophore constructed from non-covalent assembly of a G-quadruplex and liponucleoside transports K^+^-ion across biological membranes

**DOI:** 10.1038/s41467-019-13834-7

**Published:** 2020-01-24

**Authors:** Manish Debnath, Sandipan Chakraborty, Y. Pavan Kumar, Ritapa Chaudhuri, Biman Jana, Jyotirmayee Dash

**Affiliations:** School of Chemical Sciences, Indian Association for the Cultivation of Science, Jadavpur, Kolkata, 700032 West Bengal India

**Keywords:** Chemical biology, Chemistry

## Abstract

The selective transport of ions across cell membranes, controlled by membrane proteins, is critical for a living organism. DNA-based systems have emerged as promising artificial ion transporters. However, the development of stable and selective artificial ion transporters remains a formidable task. We herein delineate the construction of an artificial ionophore using a telomeric DNA G-quadruplex (*h-TELO*) and a lipophilic guanosine (**MG**). **MG** stabilizes *h-TELO* by non-covalent interactions and, along with the lipophilic side chain, promotes the insertion of *h-TELO* within the hydrophobic lipid membrane. Fluorescence assays, electrophysiology measurements and molecular dynamics simulations reveal that **MG**/*h-TELO* preferentially transports K^+^-ions in a stimuli-responsive manner. The preferential K^+^-ion transport is presumably due to conformational changes of the ionophore in response to different ions. Moreover, the ionophore transports K^+^-ions across CHO and K-562 cell membranes. This study may serve as a design principle to generate selective DNA-based artificial transporters for therapeutic applications.

## Abstract (web summary)

DNA based ionophores can mediate efficient ion transport and are crucial to understand the biological role of their natural counterparts. Here authors engineer a telomeric DNA G-quadruplex based ionophore which transports K^+^ in a stimuli responsive manner.

## Introduction

The transport of ions across the semi-permeable cell membrane is a fundamental biological process that play key roles in signal transduction, nerve impulse, muscle contraction, hormonal regulation, and apoptosis^1,2^. Dysfunctional ion transport activity may cause Alzheimer's disease, Parkinson's disease, cystic fibrosis, vision disorders etc.^3^. The naturally occurring ion channels selectively transport a particular type of metal ions across the cellular membrane. To mimic the function of these protein-based ion channels, artificial ionophores are being developed. Several synthetic scaffolds^4–13^, polypeptides^14^, nanopores^15,16^, and even DNA-based materials^17–20^ have been reported^21–26^. Specifically, the idea of using DNA to imitate the activity of membrane-protein functions has generated wide attention due to their enhanced ability to form predetermined structures with predictable mechanical properties^27–30^. Membrane-spanning DNA ionophores of different dimensions have been constructed using a single duplex DNA^31^ or programmed assembly of multiple duplex DNA sequences^32^. These DNA-based ionophores can mediate efficient ion transport and hence they are crucial to understand the biological role of their natural counterparts^33^. In addition, ionophores find important applications in a wide range of therapeutics (antibiotics, antimicrobials etc.)^34,35^, DNA sequencing, sensors^36,37^, and delivery systems^38^. Unlike natural ion channels, artificial ionophores show low selectivity and lack stimuli-responsive behavior. Therefore, developing artificial ionophores with preference for a particular ion is an attractive area of research^39–42^.

In this backdrop, cation-rich four stranded G-quadruplex DNA^43^ presents an ideal scaffold to construct cation-selective ionophores. The basic structural unit of a G-quadruplex is G-quartet, made of four guanines associated by Hoogsteen type of hydrogen bonding. The G-quartets stack upon each other to form G-quadruplex, which is further stabilized by metal ions (e.g., K^+^, Na^+^ etc.). Synthetic guanosine derivatives that can self-assemble to form columnar assemblies of G-quartets have been reported to transport cations across lipid bilayers^44–48^. In contrast to synthetic guanosine derivatives, the exterior of a G-quadruplex DNA contains negatively charged phosphate backbone. As a result, the insertion of a charged G-quadruplex structure in the hydrophobic lipid membrane is energetically unfavorable. We hypothesized to use non-covalent assembly of a G-quadruplex with a lipophilic ligand to construct artificial ionophores. This method excludes the rigorous development of covalently linked lipophilic DNA molecules for the insertion into the membrane.

**Fig. 1:**
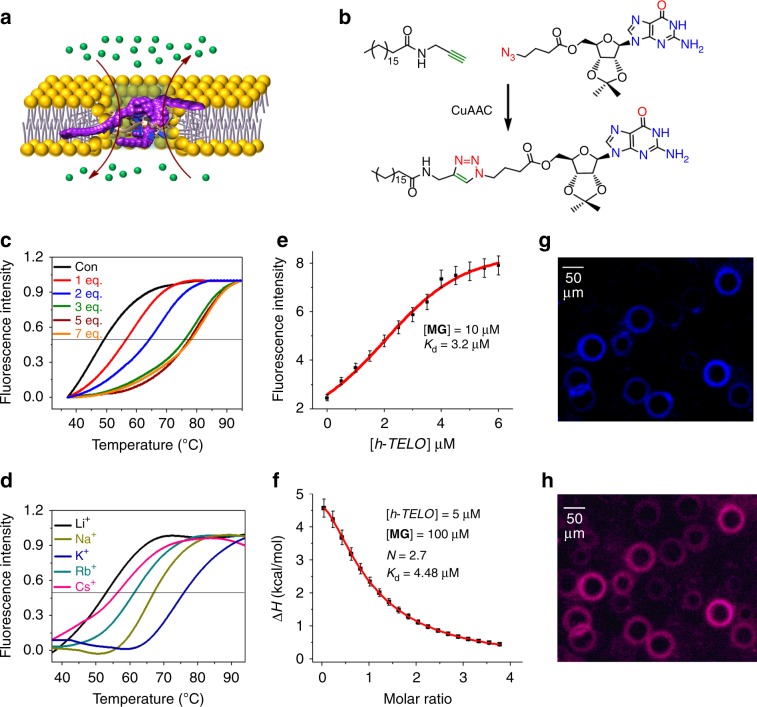
Interaction of **MG** with *h-TELO*. **a** Scheme showing K^+^ transport by the *h-TELO* ionophore within a lipid membrane. The *h-TELO* G-quadruplex is anchored to the lipid membrane by guanosine derivatives (**MG**) containing lipophilic chains (purple). The K^+^-ions are depicted as green balls and Cl^−^-ions are shown as orange balls. **b** Scheme outlining the synthesis of monoguanosine derivative (**MG**). **c** Melting profile of *h-TELO* stabilized by increasing concentration (0–7 equiv.) of **MG** in 10 mM HEPES, 100 mM KCl buffer, pH 7.4 (T_m_ of *h-TELO* is 49 °C in the same buffer). **d** Melting profile of 3:1 **MG/***h-TELO* in the presence of different metal ion containing buffer (10 mM HEPES, pH 7.4, containing 100 mM LiCl, NaCl, KCl, RbCl, and CsCl, respectively). **e** Fluorescence intensity profile of **MG** upon titration with increasing concentration of *h-TELO* in 10 mM HEPES, 100 mM KCl buffer, pH 7.4. **f** ITC titration of **MG** with *h-TELO* in 10 mM HEPES, 100 mM KCl buffer, pH 7.4. Fluorescent images (*λ*_em_ = 355 nm) of lipid vesicles (GUV) **g** with **MG/***h-TELO* and **h** co-staining with **MG/***h-TELO* and membrane-binding dye, Nile red. Source data are provided as a Source Data file. Data are means ± SD (*n* = 3).

In our approach, an endogenous human telomeric G-quadruplex DNA (*h-TELO*; PDB: 1KF1)^49^ is inserted into the lipid membrane by a synthetic guanosine-derivative **MG** containing a lipophilic side chain (Fig. 1a)^50,51^. The potential of **MG**/*h-TELO* ionophore to transport K^+^ in a stimuli-responsive manner is validated using fluorescence-based vesicle assay, voltage–clamp conductance measurements, and MD simulations. Finally, we shed light on ion transport across the cell membrane and the probable mechanism of ion transport under external conditions.

## Results

### Synthesis of lipophilic guanosine MG

The lipoguanosine **MG** containing a 17-carbon long lipophilic side chain was synthesized using Cu(I)-catalyzed azide−alkyne 1,3-dipolar cycloaddition of an azido functionalized guanosine and an alkyne functionalized stearic acid. The reaction was carried out using Na-ascorbate and CuSO_4_·5H_2_O in *t*-BuOH/H_2_O (1:1) to obtain the triazole-linked lipophilic guanosine derivative **MG** in 52% yield (Fig. 1b; Supplementary Fig. 1).

### Binding studies of MG with *h-TELO* G-quadruplex

First, we investigated the interaction of **MG** with *h-TELO* G-quadruplex using melting analysis by Förster resonance energy transfer (FRET)^52^, fluorometric titrations, and Isothermal calorimetry (ITC). We used a 22-mer *h-TELO* sequence that folds into a G-quadruplex in K^+^-buffer (PDB ID: 1KF1). FRET-based melting profiles of the dual-labeled (FAM and TAMRA at 5′- and 3′-ends, respectively) *h-TELO* G-quadruplex were monitored in the presence of incremental concentrations of **MG** (0–7 equiv.). Upon addition of **MG**, *h-TELO* showed an increase in melting temperature (*T*_m_) with saturating Δ*T*_m_ values (Δ*T*_m_ = 27 °C; *T*_m_ = 76 °C) at 3 equiv. ligand concentrations, suggesting that **MG** could considerably stabilize *h-TELO* G-quadruplex (10 mM HEPES, 100 mM KCl, pH 7.4) (Fig. 1c). The melting analysis of **MG/***h-TELO* in the presence of different alkali metal cations (Li^+^, Na^+^, K^+^, Rb^+^, and Cs^+^) revealed that **MG/***h-TELO* exhibited the highest *T*_m_ value in K^+^-ions (*T*_m _= 76 °C) compared to other ions (${T}_{\mathrm{m}}^{{\mathrm{Na}}}$ = 67 °C; ${T}_{\mathrm{m}}^{{\mathrm{Li}}}$ = 52 °C; ${T}_{\mathrm{m}}^{{\mathrm{Cs}}}$ = 55 °C; ${T}_{\mathrm{m}}^{{\mathrm{Rb}}}$ = 59 °C) at 3 equiv. ligand concentration (Fig. 1d).

Next, fluorescence studies revealed that **MG** is weakly fluorescent. When **MG** (10 μM) (*λ*_ex_ = 265 nm) was titrated with *h-TELO* (0–6 μM) in 100 mM KCl, 10 mM HEPES at pH 7.4, a dose-dependent increase in fluorescence intensity (*λ*_em_ = 355 nm) of **MG** was observed (Fig. 1e; Supplementary Figs. 2, 3). The dissociation constant (*K*_d_) was calculated to be 3.2 μM. The Job’s plot analysis revealed that **MG** binds to *h-TELO* with a 3:1 stoichiometry (Supplementary Fig. 4). The ITC data further suggested a 3:1 binding stoichiometry between **MG** and *h-TELO* (*K*_d_ = 4.48 μM) (Fig. 1f; Supplementary Fig. 5).

### Membrane localization of MG/*h-TELO*

As **MG** showed “turn-on” fluorescence upon binding to *h-TELO*, we employed fluorescent imaging to examine the localization of **MG**/*h-TELO* in giant unilamellar vesicles (GUV, lipid bilayer). GUVs were prepared using a 9:1 mixture of lipid and cholesterol. Upon addition of 20 μM **MG/***h-TELO*, blue rings were observed around the GUVs. Interestingly, these blue rings were co-localized with the membrane staining dye Nile red, indicating that **MG**/*h-TELO* is inserted in the membrane (Fig. 1g, h). However, no fluorescence was observed when GUVs were treated with either *h-TELO* or **MG**. Thus, the insertion of **MG**/*h-TELO* within lipid bilayer could be detected without using fluorescent labels.

### Ion-transport activity of MG/*h-TELO* by fluorescence assay

**Fig. 2:**
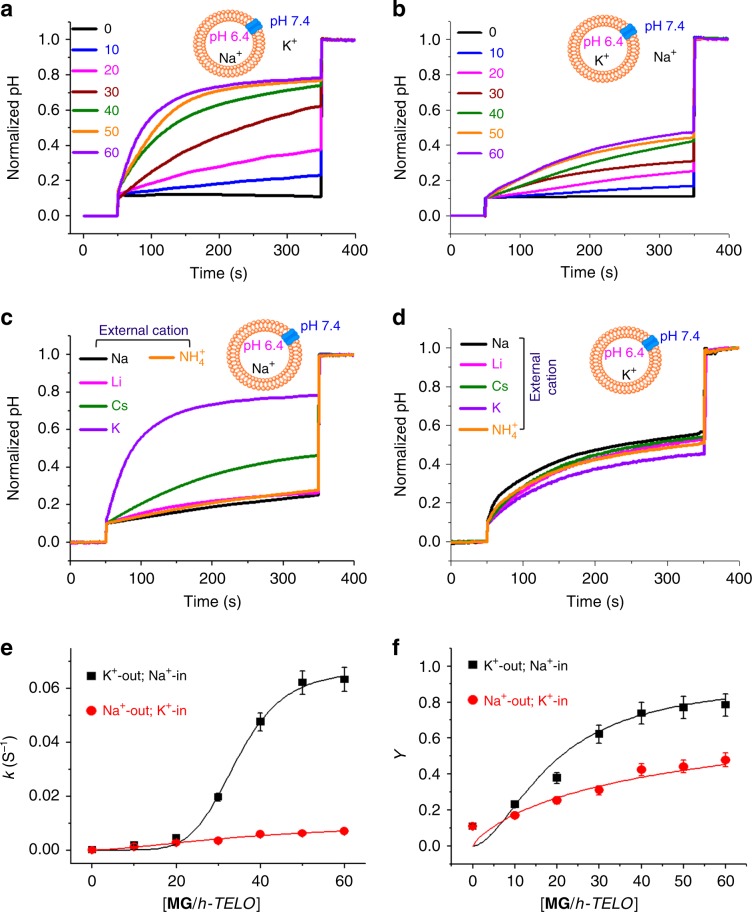
Fluorescence-based ion-transport assay. Change in normalized pH inside LUV (pH_in_) as a function of time with increasing addition of **MG**/*h-TELO* (0–60 μM) in the presence of different metal ions and pH gradient. **a** Buffer composition, external: 10 mM HEPES, 100 mM KCl, pH 7.4; internal: 10 mM HEPES, 100 mM NaCl, pH 6.4. **b** Buffer composition, external: 10 mM HEPES, 100 mM NaCl, pH 7.4; internal: 10 mM HEPES, 100 mM KCl, pH 6.4. Change in normalized pH inside LUV as a function of time in the presence of **MG**/*h-TELO* (60 μM) using different external metal ions. **c** Buffer composition, external: 10 mM HEPES, 100 mM MCl (M = Na^+^, Li^+^, Cs^+^, NH_4_^+^, and K^+^), pH 7.4; internal: 10 mM HEPES, 100 mM NaCl, pH 6.4. **d** Buffer composition, external: 10 mM HEPES, 100 mM MCl (M = Na^+^, Li^+^, Cs^+^, NH4^+^, and K^+^), pH 7.4; internal: 10 mM HEPES, 100 mM KCl, pH 6.4. **e** Pseudo-first-order initial constant rate as a function of final concentration of **MG**/*h-TELO* in the bilayer membrane. **f** Fractional activity Y = f([**MG**/*h-TELO*]) plotted for Na^+^ and K^+^ ions and the curves are the best fit from the Hill equation. Source data are provided as a Source Data file. Data are means ± SD (*n* = 3).

To evaluate the transport of alkali cations across the lipid bilayer, a fluorescence-based assay was performed using 8-hydroxypyrene-1,3,6-trisulfonic acid (HPTS) dye^53^ (Fig. 2). Liposomes (large unilamellar vesicles (LUV), 100 nm diameter) were filled with a pH-sensitive dye HPTS (*λ*_ex_ = 460 nm, *λ*_em_ = 510 nm) in 100 mM NaCl and HEPES buffer solution (10 mM, pH 6.4). The liposomes were then suspended in an external HEPES buffer solution (10 mM, pH 6.4) containing 100 mM KCl. A pH gradient, ΔpH = 1 (internal pH, pH_in_ = 6.4; outside pH, pH_out_ = 7.4) across the lipid bilayer was applied by the addition of NaOH. The formation of an ionophore by **MG**/*h-TELO* assembly was expected to induce a pH gradient collapse via H^+^ efflux leading to an increase in the pH_in_ of the vesicles. This efflux of H^+^ ions triggers an influx of external cation (K^+^, Na^+^ etc.) to maintain the equilibrium of electrical charge across the lipid bilayer. Complete destruction of the pH gradient was achieved by addition of 10% Triton X-100. The change in pH_in_ of the vesicles was monitored by the change in the fluorescence of HPTS, which indicates cation transport across the lipid bilayer. The relative pH change was quantified upon addition of **MG**/*h-TELO* in the presence and absence of external pH gradient and subsequently the rate constants were calculated as described in Supplementary Information (Supplementary Fig. 6).

For each batch of experiments, a blank experiment in the absence of additives demonstrated that a basal level change in pH_in_ was observed due to simple diffusion through the liposome bilayer. To exclude the possibility of vesicle rupture or fusion as a probable cause of change in pH_in_ values, dynamic light-scattering studies were carried out at different time points. The uniformity of vesicle size at all time points confirmed that no fusion or rupture of vesicles took place upon addition of **MG**/*h-TELO* (Supplementary Fig. 7). Additional control experiments further revealed that there were insignificant changes in pH_in_ either with free **MG** or *h-TELO* (Supplementary Fig. 8). The addition of **MG/***h-TELO* (60 μM) generated a rapid increase in pH_in_, indicating a fast efflux of proton and a high-influx rate for the external K^+^-ions (external buffer: 10 mM HEPES, 100 mM KCl, pH 7.4) (Fig. 2a). However, when the external and internal buffers were reversed maintaining the pH gradient (pH_in_ 6.4 and pH_out_ 7.4), significantly slow change in internal pH was observed (Fig. 2b).

To investigate whether the change in pH_in_ is dependent on concentration of **MG/***h-TELO*, we performed concentration-dependent HPTS assay. Upon incremental addition of **MG/***h-TELO* (0–60 μM), a dose-dependent increase in pH_in_ (external buffer: 10 mM HEPES, 100 mM KCl, pH 7.4; internal buffer: 10 mM HEPES, 100 mM NaCl, pH 6.4) was observed which attained saturation at 50 μM concentration (Fig. 2a). In contrast, **MG/***h-TELO* (60 μM) showed significantly lower activity when external KCl was replaced with NaCl (internal buffer: 10 mM HEPES, 100 mM KCl, pH 6.4) (Fig. 2b). Using other alkali metal chlorides (LiCl and CsCl) and ammonium ions (NH_4_^+^) in external buffer, minimal changes in internal pH were observed (Fig. 2c). In addition, lucigenin assay suggested that **MG/***h-TELO* could not transport Cl^−^-ions (Supplementary Fig. 9). Furthermore, when 100 mM KCl was present in internal solution, a small change in internal pH, irrespective of different metal ions in external buffer was observed (Fig. 2d). These experiments suggest that ionophores formed by **MG/***h-TELO* are responsive to K^+^-ions.

The transportation activity for Na^+^ and K^+^ was then investigated by varying the concentration of **MG/***h-TELO* from 0 to 60 μM. **MG/***h-TELO* exhibited pseudo-first-order kinetics for the transport of both cations (Supplementary Figs. 10, 11). Rate constant (*k*_obs_) values were plotted against increasing concentrations of **MG/***h-TELO* (Fig. 2e). The transport rates for K^+^ (*k*_K_^+^) were found to be saturated at 50 μM **MG/***h-TELO* concentration, whereas no saturation in transport rates for Na^+^-ions (*k*_Na_^+^) was obtained. At this concentration (50 μM), a ~10-fold higher transport rate for K^+^-ions (*k*_K_^+^ = 0.062 s^−1^) was observed over Na^+^ (*k*_Na_^+^ is 0.0062 s^−1^). To determine the effective concentration of **MG/***h-TELO* required to obtain 50% of transport activity (EC_50_) and the stoichiometry of the transport process (Hill coefficient), the fractional activity at 300 s for each concentration was plotted against the concentration of **MG/***h-TELO* (Fig. 2f). Hill analysis of the plots revealed that the EC_50_ values for K^+^ and Na^+^ were 19 μM and 54 μM, respectively. The Hill coefficients (*n*) for both cations were also found to be different: 1.76 for K^+^ and 0.81 for Na^+^. Together, these results indicate that ionophores formed by **MG**/*h-TELO* preferentially transport K^+^-ions over other alkali metal ions.

Next, the ability of **MG/***h-TELO* to transport cations in the absence of pH gradient (passive transport) was investigated using HPTS assay (Supplementary Figs. 12, 13). Interestingly, this passive transport was activated only in the presence of cation concentration gradient across the lipid bilayer. In the presence of same internal and external buffer (containing 100 mM NaCl, pH 6.4), a negligible change in the internal pH was observed upon treatment with **MG/***h-TELO* (60 μM) (*t* = 50 s), indicating no passive transport.

However, when KCl was present in external buffer, passive transport was observed in a dose-dependent manner by addition of **MG/***h-TELO* (60 μM) (*t* = 50 s). Interestingly, when KCl was present inside the vesicle (NaCl outside), passive transport was reversed. These results suggest that passive ion transport is dependent on the concentration gradient of K^+^-ions. In addition, safranin assay suggested that the passive ion transport by *h-TELO* ionophore can alter the transmembrane potential (Supplementary Fig. 14).

### Atomistic insight into the formation of MG/*h-TELO* ionophore

**Fig. 3:**
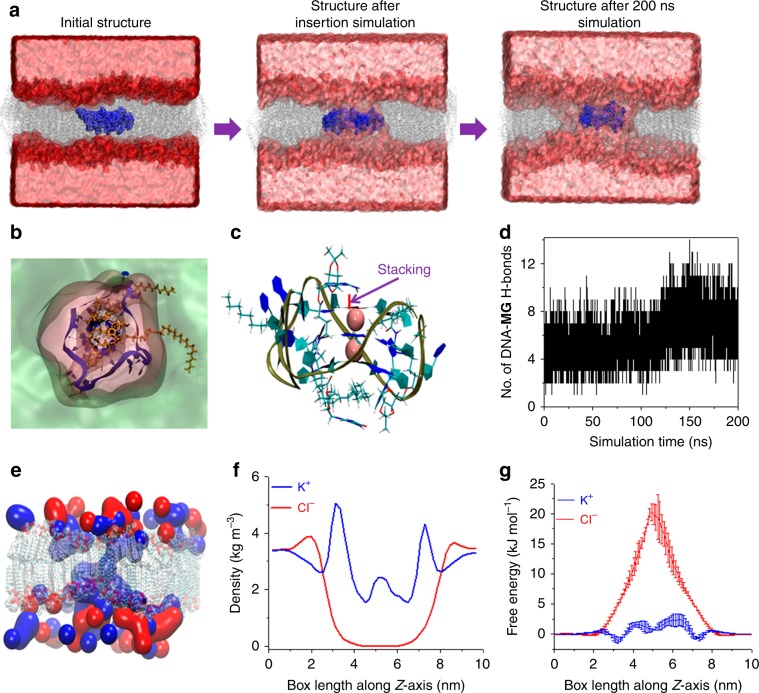
MG/*h-TELO* ionophore formation within lipid bilayer. **a** Process of pore formation around *h-TELO* within the lipid membrane. Initially, ligand-bound quadruplex (blue vdW representation) is placed within the lipid bilayer (gray stick) in a water box (red surface). **b** Final structure of the supramolecular quadruplex-based ion pore. Lipid bilayer is colored in green surface, whereas the water pore surrounding the quadruplex is shown in pink surface. *h-TELO* is shown in blue cartoon, and bound **MG** is shown in orange sticks. **c** Structure of **MG/***h-TELO* is shown. G-quadruplex is shown in cartoon representation, whereas bound ligand is shown in stick mode. **d** The number of hydrogen bonds between *h-TELO* and **MG** during the simulation of the ionophore is shown. **e** A representative snapshot of K^+^ (blue) and Cl^−^ (red) ion distributions in the simulation box is shown. Lipid bilayer is shown as gray transparent surface. **f** Density profiles of K^+^(blue) and Cl^-^(red) ions averaged over the entire 200 ns simulation trajectory are shown. **g** Potential of mean force profile for K^+^ (blue) and Cl^−^ (red) translocation across the simulation box (*Z*-axis). Source data are provided as a Source Data file. Data are means ± SD (*n* = 3).

Equilibrium simulations were then performed to gain atomistic insights into the formation of ionophore in the membrane (Fig. 3). Simulations were performed by using parmbsc1 force field^54^ implemented in GROMACS simulation packages^55,56^. The details of the simulation methodologies and validations of the force field for G-quadruplex and membrane simulations have been provided in the Supplementary Information (Supplementary Figs. 15, 16). Equilibrium simulations of **MG**/*h-TELO* with different stoichiometries (1:1, 2:1, & 3:1) revealed that native G-quadruplex shows the highest stabilization in the presence of three molecules of docked **MG**, characterized by low root-mean-square deviation from the crystal structure throughout the simulation trajectory (Supplementary Fig. 17). This result complements FRET-based melting and isothermal calorimetry studies. During equilibrium simulation of **MG/***h-TELO* with a 3:1 stoichiometry, guanosine moieties of **MG** stack on the terminal quartet of G-quadruplex, while the long alkyl tails wrap the quadruplex along the groove region to form a highly stable assembly (Supplementary Fig. 18). **MG/***h-TELO* was then inserted within the membrane such that the K^+^-filled pore of the *h-TELO* was aligned along the *Z*-axis. The structure of the initial assembly is shown in Fig. 3a. During membrane insertion simulation, water molecules from the bulk came toward the *h-TELO* and hydrated the quadruplex structure within the lipid membrane. The **MG/***h-TELO* subsequently underwent large conformational transition to form highly stable ionophore. Once, water-filled pore was formed inside the membrane, the lipophilic chains of **MG** in the *h-TELO* were incorporated into the hydrophobic region of the lipid bilayer constituted by the fatty acid tails of phospholipid. Interestingly, among the three bound **MG** molecules, two of them anchor the *h-TELO* with the membrane, the third one wraps the DNA along the groove and its guanosine moiety perfectly stacks with the G-quartet providing additional stability to the G-quadruplex inside the pore. The charged phosphate backbone of the G-quadruplex was thus exposed within the membrane, which attract more and more water molecules from the bulk to solvate the G-quadruplex to form a water-filled channel within the membrane. Adjacent phospholipids also underwent structural rearrangements such that their phosphate head groups could face toward the water pore. This assembly was found to be highly stable throughout simulation. The formation of such water channel along the simulation trajectory is shown in Fig. 3a. Upper view of the supramolecular ionophore is shown in Fig. 3b. **MG** molecules played crucial role in stabilizing the *h-TELO*, apart from anchoring it within the membrane matrix. The guanosine moiety stabilized the G-quartet through stacking interactions (Fig. 3c), and other polar groups of **MG** could form ~6–8 hydrogen bonds with the *h-TELO* (Fig. 3d). In a control simulation using only *h-TELO*, it was observed that in the absence of **MG**, *h-TELO* diffused away from the water pore (Supplementary Fig. 19). Interestingly, the translational diffusion of the *h-TELO* increased in the absence of **MG** and it could not retain the G-quadruplex structure in the membrane. The stacking of G-quartets in the G-quadruplex was observed to be lost during the simulation timescale (Supplementary Figs. 19, 20).

From equilibrium simulation, we further monitored the distribution of K^+^ and Cl^−^ ions by inserting **MG**/*h-TELO* in the lipid membrane in 200 mM KCl solution. A representative snapshot of K^+^ (blue) and Cl^−^ (red) ion distribution in the simulation box is shown in Fig. 3e, and the corresponding density profiles of the two ions averaged over the entire 200 ns simulation trajectory are shown in Fig. 3f.

As evident from the figure, K^+^-ions were preferentially distributed around the phosphate groups of the bilayer. In addition, there was significant K^+^-ion density within the water pore. The peak in the K^+^-ion density profile within the water pore corresponded to its preferential distribution along the phosphate backbone of the G-quadruplex. In contrast, Cl^−^ was distributed mostly in the bulk with no noticeable density within the water pore due to electrostatic repulsion from the negatively charged phosphate backbone of *h-TELO*. The potential of mean force calculation revealed that the free-energy of K^+^-ion movement from the bulk to the water pore was within the diffusion control limit, whereas free-energy barrier for the same translocation of a Cl^−^-ion was ~10 times higher (Fig. 3g).

### Voltage-dependent ion-transport induced by the MG/*h-TELO*

**Fig. 4:**
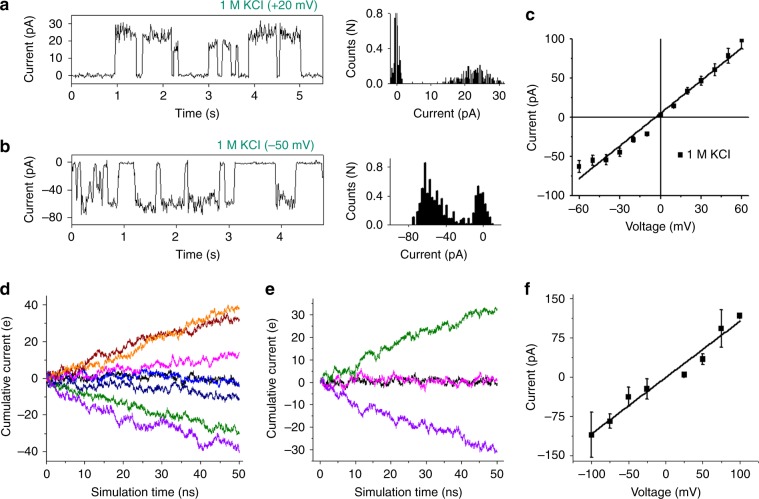
Ion conductance studies across the lipid bilayer by MG/*h-TELO*. Single-channel current traces recorded at +20 mV (**a**) and −50 mV (**b**) holding potentials in 1 M symmetrical KCl solution in the presence of **MG/***h-TELO* (60 μM). All point histograms generated from the corresponding current traces at +20 mV and −50 mV have been presented on the right. **c** *I*−*V* plot using a voltage ramp (−60 mV to +60 mV) in 1 M symmetrical KCl solution (black line) in the presence of **MG/***h-TELO* (60 μM). Data are means ± SD (*n* = 3). **d** Cumulative currents through the transmembrane pore resulting from the application of external electric fields. Orange, maroon, pink, black, light blue, deep blue, green, and purple color represent +100 mV, +75 mV, +50 mV, +25 mV, −25 mV, −50 mV, −75 mV, and −100 mV applied voltage biases. **e** Cumulative current flow for K^+^ ions under the applied voltage biases of +100 mV (green), −100 mV (purple) and for Cl^−^ ions under the applied voltage biases of +100 mV (black), −100 mV (pink). **f** Current/voltage characteristics of supramolecular transmembrane ionophore computed from molecular dynamics simulations under different applied voltage biases. Source data are provided as a Source Data file. Data represented as Mean ± SD (*n* = 4).

To investigate voltage-dependent K^+^-ion transport activities of **MG/***h-TELO* across the lipid membrane; ionic current recording studies were conducted as follows: phospholipid bilayers with 1 M KCl containing buffer at the *cis* and *trans* chambers were prepared, and the current was recorded after addition of **MG/***h-TELO* (60 μM) to the *cis* chamber. Interestingly, we observed transient fluctuations between distinct current values. From the histogram, it is clear that the current fluctuation corresponds to two states of the ionophore: a fixed closed state (mostly around 0 pA) and variable open states depending upon the sign and magnitude of the holding potential (Fig. 4a, b). The conductance values are calculated to be 1.6 ± 0.4 nS in the presence of 10 mM HEPES buffer containing 1 M KCl (pH 7.4). In addition, **MG/***h-TELO* exhibited significantly high transport activity with long opening periods at +80 mV holding potential (Supplementary Fig. 21). However, no fluctuation in current was observed in control experiments using  either **MG** or *h-TELO* (Supplementary Fig. 22). When KCl was replaced with NaCl in the buffer, **MG*/****h-TELO* exhibited considerably weaker transport activity (Supplementary Fig. 21). Other chlorides like LiCl, RbCl, CsCl, and NH_4_Cl showed negligible transport activity (Supplementary Fig. 23).

In order to understand the K^+^-ion transport property, a current−voltage relationship (*I*−*V* plot) was further derived (Fig. 4c). The *I*−*V* plot follows an ohmic relation with symmetrical currents at negative and positive polarity ranging from −50 mV to +50 mV. The linear fit of the plot showed a slope value of 1.4 nS.

### Atomistic insight into the ion-transport by MG/*h-TELO*

Ion conductance simulations were next carried out to provide atomistic insight into the ion transport across the lipid bilayer through the ionophore formed by **MG**/*h-TELO*. NPzAT simulations were carried out in 200 mM KCl solution, in line with fluorescence-based ion-transport assay, to measure current flow in the presence of an external electric field along the *Z*-axis. The system was simulated at +100, +75, +50, +25, −25, −50, −75, and −100 mV transmembrane biases for ∼80 ns at each bias. During initial simulation phases, ions and water molecules in the bulk rapidly reoriented under the influence of an external electric field and attained stationary phase, where the applied electric field essentially generated transmembrane voltage bias. Therefore, initial 30 ns of simulation trajectories were not considered during cumulative current calculations to ensure that all the systems attain stationary state. Cumulative current was conducted by a method originally introduced by Aksimentiev et al.^57^, and later classified as total intensity method by Faraudo et al.^58^. Evident from Fig. 4d, the cumulative total current flow linearly increased with simulation time under the effect of applied voltage which implicate that all the system attained stationary state. We then calculated the contribution of K^+^ and Cl^−^ on total current flux. In all applied voltage bias, the current conducted through the system was contributed from the K^+^-ion translocations only. Current conducted by the Cl^−^ion was negligible in all applied voltage bias indicated its movement was primarily diffusion controlled and unaffected by the transmembrane voltage bias (Fig. 4e). Therefore, Cl^−^ could not penetrate the transmembrane pore, in line with equilibrium simulation and experiments. Calculated I−V plot (Fig. 4f) from the simulation within +100 mV to – 100 mV region was found to be linear thus follow ohmic relation. The average conductance calculated from the simulation was 1.075 nS, which is in excellent agreement with the conductance obtained from voltage–clamp experiments.

### Proposed mechanism for ion transport by the MG/*h-TELO*

**Fig. 5:**
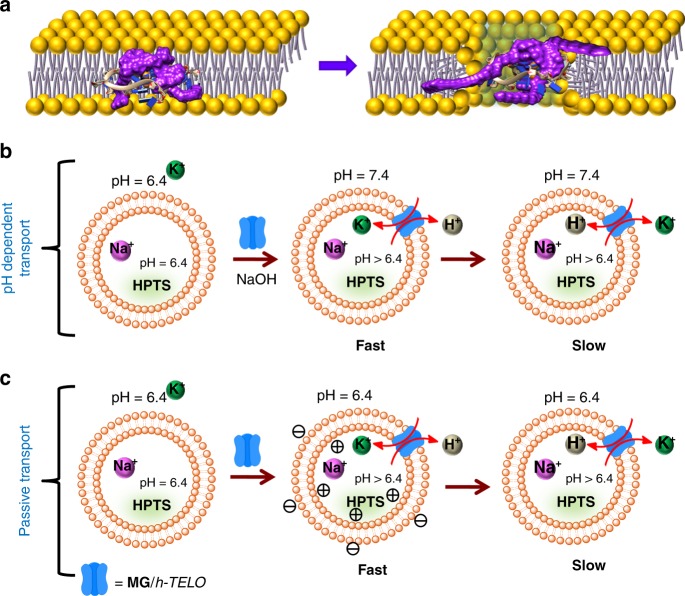
Proposed mechanism for ion transport in fluorescence assay. Schematic representation showing **a** the formation of water-filled ionophore in the lipid bilayer by rearrangement of lipid molecules upon insertion of **MG**/*h-TELO*. The lipophilic chains of **MG** anchors the **MG**/*h-TELO* to stabilize the ionophore. **b** The stepwise ion transport (left to right) by pH-dependent transport (top) and; **c** passive diffusion (bottom).

The plausible mechanism for the cation transport (K^+^) in lipid vesicles by **MG**/*h-TELO* with pH gradient is interpreted based on the experimental data (Fig. 5). Initially, Na^+^ ions filled vesicles are placed in K^+^-ion buffer to create an ion gradient across the lipid bilayer. The addition of **MG**/*h-TELO* induces rapid reorientation of phosphate head groups in the bilayer to form ionophores (Fig. 5a). A pH gradient across the lipid membrane of the vesicles (pH_in_ = 6.4 and pH_out_ = 7.4) is induced by adding NaOH in the medium. This triggers a H^+^ efflux, followed by a K^+^ influx down the concentration gradient (to maintain the charge equality) showing hyperbolic increase in pH_in_ values with time (Fig. 5b). The plateau region is reached due to a slow H^+^/K^+^ exchange that equillibrates the K^+^-ion concentration and pH inside the vesicle (small amounts of Na^+^ efflux may also take place down the concentration gradient). Importantly, when **MG** was heat annealed in K^+^ solution and added to the vesicles, no significant ion transport was observed. This is in accordance with CD studies which show that **MG** has a very low efficiency to form G-quadruplex like structures compared with diguanosine derivatives^44^ reported before (Supplementary Fig. 24). The addition of Triton X solubilizes lipid membranes to finally equilibrate the ionic concentration and pH in the entire solution. In the presence of Na^+^ in external buffer, small changes in pH_in_ values suggest that lower influx of Na^+^ possibly retards H^+^ efflux. In addition, the presence of other alkali cations (Li^+^, NH_4_^+^, and Cs^+^) in external buffer shows negligible changes in pH_in_ values, suggesting their reduced influx. In passive diffusion experiments (K^+^ outside and Na^+^ inside), **MG**/*h-TELO* activates K^+^ influx down the concentration gradient along with a H^+^ efflux, and membrane polarization takes place (observed from Safranin O assay) as sharp increase in pH_in_ values are observed (Fig. 5c). When the internal and external buffer is exchanged (K^+^ inside and Na^+^ outside), a fast K^+^ efflux followed by a H^+^ influx takes place as evidenced by a sharp decrease in pH_in_ values. However, the changes in pH_in_ values are much higher by applying external pH gradient.

The nature of ion transport by **MG**/*h-TELO* has been elucidated by HPTS, Lucigenin, Voltage clamp and MD simulation studies. The MD simulation studies along with the negative Lucigenin assay data strongly suggest that the conducting ions are cationic metal ions and not anionic Cl^−^ions. This may be due to the presence of negatively charged phosphate backbone of G-quadruplex which repels the anions from entering the water-filled channel.

### MG/*h-TELO* as K^+^ ionophore

To decipher the origin of the high transport rates of **MG**/*h-TELO* ionophore for K^*+*^ ions, we examined three systems: K^+^-stabilized **MG**/*h-TELO* in KCl and NaCl media, respectively, and Na^+^-stabilized **MG**/*h-TELO* in NaCl media.The systems were simulated using same protocol with the applied external electric field of +75 mV and −75 mV and ion-conductance values were calculated.

**Fig. 6:**
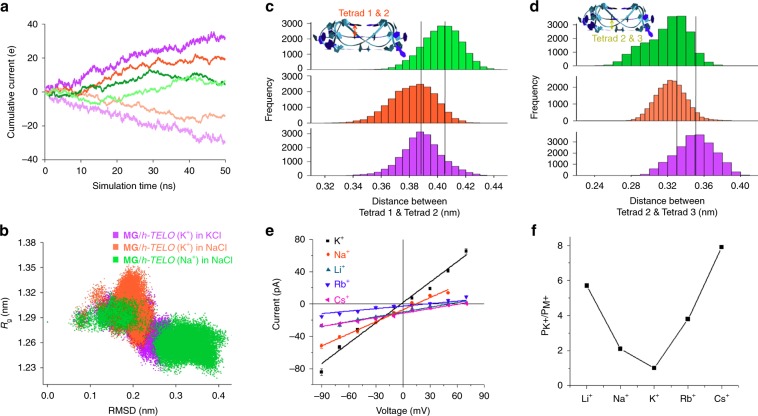
MG/*h-TELO* as K^*+*^ ionophore. Molecular dynamics simulations of **MG**/*h-TELO* in KCl and NaCl electrolyte solutions, represented by purple and orange color, respectively. The Na^+^-stabilized **MG**/*h-TELO* in NaCl electrolyte solution is represented by green color. **a** Cumulative current flow computed for the three systems under the applied voltage biases of +75 mV, rendered in the dark color and −75 mV, represented in light color. **b** 2-D scatter plot of root-mean-square deviation (RMSD) and radius of gyration (R_g_) obtained from the equilibrium simulations. Variations of the distance vector along the *Z*-axis between tetrads 1 & 2 **c** and tetrads 2 & 3 **d** are shown for **MG**/*h-TELO* ionophore embedded in the lipid bilayer in three different conditions obtained from the simulations. **e** I–V curves generated from the tail currents of the traces in different buffer conditions. Bi-ionic conditions were used: 10 mM HEPES buffer containing 0.150 M KCl in the *cis* side and 0.150 M different metal chlorides (LiCl, NaCl, KCl, RbCl, and CsCl) in the *trans* side. **f** Permeability ratios of **MG**/*h-TELO* obtained for different monovalent cations with respect to K^+^ ion. Source data are provided as a Source Data file. Data represented as mean ± SD (*n* = 4).

These studies indicate that the K^+^-stabilized **MG**/*h-TELO* ionophore exhibits higher flow of cumulative current for KCl over NaCl solution. In KCl solution, the cumulative total current flow linearly increases with simulation time under the effect of applied voltage in either direction, implicating the system attained stationary state and there is steady flow of current (Fig. 6a). The K^+^-stabilized **MG**/*h-TELO* may transport different monovalent ions, depending on the mobility of the ions. However, for Na^+^-stabilized **MG**/*h-TELO*, steady flow of current is not observed, and stationary state is not attained.

Next, the structure of the **MG**/*h-TELO* ionophore in these systems was assessed from the equilibrium simulations. These results show that **MG**/*h-TELO* ionophore exists in different conformations in response to K^+^ and Na^+^ ions. As evident from the 2-D RMSD-R_g_ plot, an increase in RMSD and subsequent deviation of R_g_ are observed in Na^+^-stabilized **MG**/*h-TELO*, suggesting perturbation of the native structure with simulation time (Fig. 6b). We further quantified how different ions perturb the G-quadruplex structure by monitoring the inter-quartet distances (Fig. 6c, d). The stacking pattern remained intact, for K^+^-stabilized **MG**/*h-TELO*, as evident from comparable distance distribution. In contrast, the stacking interactions were perturbed for **MG**/*h-TELO* in Na^+^ media.The distance between Tetrad 1 and Tetrad 2 increases and the distance between Tetrad 2 and Tetrad 3 is widely distributed, indicating structural changes in the quadruplex topology in Na^+^ media. CD spectroscopy also indicates that G-quadruplex exist in different conformations in the presence of other alkali metal ions than in K^+^ (Supplementary Fig. 25). Therefore, **MG**/*h-TELO* may regulate ion transport by changing its conformation in response to different ions. The simulation data further complemented the results of the melting study which indicated that the stability of the **MG**/*h-TELO* is maximum in K^+^ media (Fig. 1d).

The measurement of reversal potentials was also performed to investigate the relative permeability of different ions in comparison with K^+^ through **MG/***h-TELO* ionophore. To stabilize the ionophores, it is important to use K^+^ containing buffer in the *cis* side of the NPC chip (external side, i.e., the side of the addition of **MG/***h-TELO*). We used a bi-ionic system of 10 mM HEPES buffer containing 0.15 M KCl in the *cis* side and 0.15 M different metal chlorides (internal side, i.e., LiCl, NaCl, KCl, RbCl, and CsCl) in the *trans*-side^59^. The holding potential was first increased to +100 mV and then switched to various testing potentials (−90 mV to +70 mV). The reversal potential was measured from an *I*–*V* curve, constructed from tail currents recorded at each potential (Supplementary Fig. 26). Under these conditions, control vesicles exhibited no change in current at different voltages. Upon addition of 60 μM **MG/***h-TELO*, different current (I) values corresponding to different voltages were recorded. The reversal potential value for 0.15 M KCl in the *trans* side showed value close to 0 mV, indicating that **MG/***h-TELO* exhibits almost equal permeability of ions (Fig. 6e, f). On the other hand, reversal potentials of +11 mV for NaCl (permeability ratio *P*_K_^+^/*P*_Na_^+^ = 2.1) and +30 mV to +67 mV were observed for 0.15 M LiCl, RbCl, and CsCl, respectively, yielding a permeability ratio (*P*_K_^+^/*P*_M_^+^) in the range of 3.8–7.9. These results strongly suggest that MG/h-TELO ionophore preferentially transports  K^+^ ions.

### Inhibition and switching activity of the ionophore

**Fig. 7:**
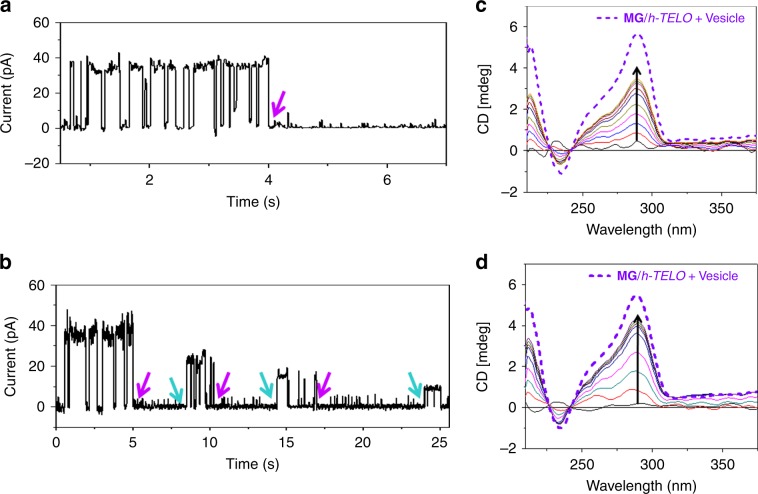
Inhibition of ion transport. Single-channel current traces recorded in the presence of **MG**/*h-TELO* (60 μM) in 1 M symmetrical KCl solution at +30 mV (**a**) showing the disappearance of ion-transport activity upon treatment with thiazole orange (TO) (4 equiv. of **MG**) (pink arrow) and **b** inhibition in ion-transport activity upon addition of cytosine (3 equiv. of **MG**) and the reversal of ion-transport activity for three cycles with addition of **MG**/*h-TELO* (2 equiv. **MG** over cytosine in each cycle, cyan arrow). CD spectra of **MG**/*h-TELO* in vesicles in 10 mM HEPES buffer containing 1 M KCl (pH 7.4) (purple) and the CD spectra of initial supernatant after separating vesicles by centrifugation (black line). Gradual increase in CD intensity upon addition of **c** TO (0–4 equiv. of **MG**) and **d** cytosine (0–3 equiv. of **MG**). Source data are provided as a Source Data file. Data are means ± SD (*n* = 3).

Since the binding of **MG** with *h-TELO* is pivotal for the formation of ionophore, the ion transportation can be inhibited by (i) the displacement of **MG** from *h-TELO* by a high affinity ligand like thiazole orange (TO) and (ii) disruption of **MG** and *h-TELO* interaction by cytosine. Upon addition of TO (60 μM), a complete disappearance of the transport activity of **MG***/h-TELO* ionophore across the lipid bilaye was observed (Fig. 7a). As TO binds to *h-TELO* with higher affinity compared with **MG** (*K*_D_ (TO) = 0.5 μM vs. *K*_D_ (**MG**) = 3.2 μM), it could displace **MG** from *h-TELO* and disrupt membrane transport by the ionophore.

A sharp decrease in conductance was also observed when 3 equiv. excess of cytosine was added to the **MG**/*h-TELO* in the lipid bilayer (Fig. 7b). Interestingly, the ionophoric activity could be triggered again by addition **MG**/*h-TELO* (2 equiv. excess of cytosine added). However, the conductance values were slightly decreased with each cycle and completely reduced after three cycles.

The release of *h-TELO* from the ionophore in the lipid bilayer by TO and cytosine was monitored by CD spectroscopy (Fig. 7c, d). The CD spectra of **MG**/*h-TELO* containing vesicles in K^+^ buffer showed a positive peak at 290 nm and a shoulder at 270 nm.

By separating the **MG**/*h-TELO* containing vesicles by centrifugation, no detectable amount of *h-TELO* in the supernatant was observed. However, the addition of aliquots of TO or cytosine to the mixture resulted in a gradual increase in the amount of *h-TELO* in the supernatant as evidenced by the appearance of the characteristic peaks for the *h-TELO*. These results suggest that TO and cytosine can disrupt the structure of the **MG**/*h-TELO* ionophore in the membrane.

### Ion-transport across the cell membrane by the MG/*h-TELO*

**Fig. 8:**
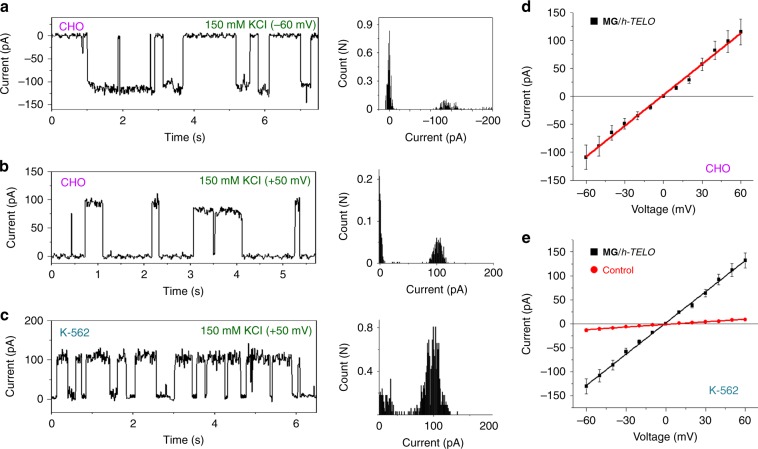
Ion conductance studies across cell membrane by MG/*h-TELO*. Single-channel current traces recorded at −60 mV (CHO cells) (**a**), +50 mV (CHO cells) (**b**), and +50 mV (K-562 cells) (**c**) in holding potentials in symmetrical solution of 10 mM HEPES, 150 mM KCl, pH 7.4 in the presence of **MG**/*h-TELO* (60 μM). All point histograms generated from the corresponding current traces at +50 mV and −60 mV have been presented on the right. **c** *I*−*V* plot using a voltage ramp (−60 mV to +60 mV) in 10 mM HEPES, 150 mM KCl, pH 7.4 symmetrical buffer in the presence of **MG**/*h-TELO* (60 μM) in CHO cells (**d**) and in K-562 cells (**e**). The red line in **e** shows the transport in control K-562 cells. Source data are provided as a Source Data file. Data are means ± SD (*n* = 20).

Voltage clamp measurements were performed to demonstrate the ability of the ionophore to transport K^+^ ions across the membranes of Chinese hamster ovary (CHO) and human erythroleukemia (K-562) cell lines (10 mM HEPES, 150 mM KCl, pH 7.4) (Fig. 8a–c; Supplementary Fig. 27). The control experiments revealed that CHO cells did not exhibit any endogenous ionophoric activity and K-562 cells showed weak ionophoric activity (conductance values in the range of 0.08 nS). Upon attaining the giga seal condition in both cell membranes, **MG**/*h-TELO* ionophore was added to the *cis* side. Under these conditions, **MG**/*h-TELO* ionophore exhibited voltage dependent K^+^ ion conductance and two distinct open and closed states across CHO and K-562 cells. The *I*–*V* plots indicated that the ionphore shows conductance values of 1.8 nS and 2.1 nS in CHO and K-562 cell membranes, respectively (Fig. 8d, e). These experiments demonstrate that the *h-TELO* ionophore can transport K^+^ across the cell membrane.

## Discussion

In this work, an ionophore is constructed from lipophilic guanosine derivative (**MG**) and *h-TELO* G-quadruplex. The resulting quadruplex DNA ionophore transports K^+^-ions across the biological membranes (Supplementary Movie 1). FRET melting, fluorescence titration, and ITC experiments establish that **MG** binds to *h-TELO* with a 3:1 stoichiometry. MD simulation studies reveal that the lipophilic chains of **MG** anchors the *h-TELO* and stabilizes  it within the lipid bilayer to form the ionophore. Fluorescence-based vesicle assays, voltage clamp, and MD simulation experiments collectively suggest that **MG***/h-TELO* ionophore preferentially transports K^+^ ions over other alkali cations. The ionophore presumably transports ions by changing its conformation in response to different ions. The reversal potential experiments suggested that the ionophore has relative permeability for K^+^ ions over different cations in the order *P*_K_^+^ > *P*_Na_^+^ > *P*_Rb_^+^ > *P*_Li_^+^ > *P*_Cs_^+^ (1.0:2.1:3.8:5.7:7.9). The ion transport can be disrupted by the addition of thiazole orange or cytosine. Interestingly, by carefully regulating the addition of **MG**/*h-TELO* and cytosine, a switch ON/OFF activity of the ionophore up to three cycles is achieved. Voltage clamp experiments demonstrate *h-TELO* ionophore transports K^+^ ions across CHO and K-562 cell membranes. Taken together, simple and viable strategy is presented to device selective DNA based ionophores, which may provide opportunities for specific targeting of cellular receptors. Furthermore, the mechanistic details of G-quadruplex-based ionophores would provide insight into controlled ion transport for biomedical applications.

## Methods

### General information

The general chemicals and DNA sequences required for biophysical analysis were purchased from Sigma-Aldrich and IDT (USA), respectively. The DNA sequences of highest purity were purchased for best results. The lipid and cholesterol were purchased from Sigma-Aldrich. The voltage clamp studies were performed using Nanion port-a-patch (Germany) system. The details for synthetic materials and procedures have been described in Supplementary Information.

### LUV preparation for HPTS experiments

Large unilamellar vesicles (LUVs) were formed using a 20 µL 9:1 mixture of 10 mM DphPC (1,2-diphytanoyl-sn-glycero-3-phosphocholine) and cholesterol in chloroform. After solvent removal and vacuum drying, the resulting thin film was hydrated with 500 µL of buffer (10 mM HEPES, 100 mM NaCl or KCl, pH 6.4) containing 10 µM HPTS (8-hydroxypyrene-1,3,6-trisulfonic acid trisodium salt). Next, the suspension was subjected to six freeze–thaw cycles (liquid nitrogen/water at room temperature) during hydration. The resulting white suspension was then extruded 19 times through a 100 nm polycarbonate membrane to obtain large unilamellar vesicles (LUVs) with an average diameter of ~100 nm. The LUVs suspension was separated from extravesicular HPTS dye by using size-exclusion chromatography (Econo-Pac 10DG column, Bio-rad; mobile phase: 10 mM HEPES, 100 mM NaCl or KCl, pH 6.4) and diluted with mobile phase to desired working concentration.

### Cation transport experiments

In a clean and dry fluorescence cuvette, 5 µL of stock HPTS containing vesicle solution was suspended in 495 µL of the corresponding buffer (10 mM HEPES, 100 mM KCl, NaCl, LiCl, NH_4_Cl, and CsCl pH 6.4). Rb salts somehow interfered with the assay and hence excluded. The fluorescence of HPTS at 510 nm was monitored upon excitation at 460 nm in a time-dependent manner. For pH gradient-mediated transport experiments, different concentrations of **MG/***h-TELO* (0, 10, 20, 30, 40, 50, 60 μM) were mixed initially (at *t* = 0 s) with vesicles. At *t* = 50 s, 7.25 µL of aqueous NaOH (0.5 M) was added, resulting in a pH increase by one unit in the extravesicular solution. Finally, at 350 s, the vesicles were lysed with detergent (10 µL of 5% aqueous Triton X-100) resulting in the destruction of pH gradient.

### Molecular dynamics simulations

All the simulations were performed using GROMACS 2016 molecular dynamics package^55,56^. A modified force field particularly developed for DNA simulation, parmbsc1^54^, and SPC/E^60^ water model was used throughout the study. Details of the system preparation, validation for the force field, and simulation methodology are provided in Supplementary Information (Molecular modeling studies, Supplementary Information). Previously, ion-channel formation by porphyrin-tagged duplex DNA was studied using atomistic simulations^31^. Motivated from the simulation protocol; here we have studied the detailed mechanism of ion-channel formation by non-covalent **MG/***h-TELO* assembly.

Briefly, parallel G-quadruplex structure of the human telomeric DNA (*h-TELO*) was obtained from the Protein Data Bank (PDB ID: 1KF1). All-atom structure of the lipid bilayer structure was obtained from the Lipidbook^61^. Slipid force field^62^ for the lipid bilayer was imported within the parmbsc1 force field and used for the simulation. Ligand (**MG**) parameters for the parambsc1 were generated using Antechamber tools^63^ and Antechamber Python Parser interface (ACPYPE)^64^.

**MG/***h-TELO* assemblies with 1:1, 2:1, and 3:1 were obtained from stepwise docking procedure using AutoDock 4.2. Each assembly was initially minimized in vacuo and then immersed into a cubic box containing SPC/E water. Appropriate numbers of K^+^ ions were added to make each system charge neutral. All the systems were energy minimized in water using steepest descent algorithm, followed by 1 ns position restrained simulation in NPT ensemble. Finally, 500 ns of production simulations were performed in NPT ensemble at 298 K by employing Berendsen thermostat, and pressure was kept constant by coupling with Berendsen barostat. Electrostatic interactions were computed using the Particle Mesh Ewald (PME) summation method.

Then a systematic procedure was followed to construct the membrane-spanning ionophore. The equilibrated bilayer was then replicated to generate a bilayer of 512 lipids. The bilayer was then solvated and subjected to 200 ns equilibrium run in NPT ensemble at 298 K. The 3:1 **MG*/****h-TELO* assembly was inserted within the large hydrated bilayer in such a way that K^+^-filled pores of the *h-TELO* were aligned along the *Z*-axis. The system was then subjected to 1 ns membrane insertion simulation using the membed protocol at 298 K. The system was then made charge neutral by adding appropriate number of K^+^ and Cl^−^ ions, such that the final salt concentration of the system became 200 mM. The system was then energy minimized, followed by 100 ns position restrained simulation in NPT ensemble at 298 K. Finally, 200 ns production run was performed in NPT ensemble. An additional system was constructed: **MG**/*h-TELO* containing Na^+^ ions and the bulk solution containing equivalent concentration of NaCl. 100 ns position restrained simulation in NPT ensemble at 298 K was performed for each system. Finally, 200 ns production run was performed in NPT ensemble for each system.

To measure the ionic current conducted by the ionophore computationally, simulations were carried out in NP_z_AT ensemble in presence of an external applied electric field (Supplementary Notes in Supplementary Information). Briefly, a voltage drop across the system was generated by applying an external electric field along the *Z*-axis. Nosé-Hoover thermostat was used to maintain temperature of the simulation at 298 K with a time constant of 0.2 ps for coupling. Area of the *X–Y* plane remains constant throughout the simulation timescale. Semi-isotropic pressure coupling was applied along the *Z*-axis using Berendsen barostat with a time constant for coupling set to 5 ps and a compressibility of 4.5 × 10^−5^ bar^−1^ along the *Z*-axis. External electric field with appropriate strength was applied along the *Z*-axis, such that the specific voltage drop was attained. We have used the total intensity method^57,58^ to compute the instantaneous current conducted by each system in presence of a voltage gradient across the system.

### Preparation of giant unilamellar vesicles (GUVs)

GUVs were prepared by electroformation technique (Vesi Prep Pro, Nanion, Germany). In total, 10 µL of a 10 mM solution of DphPC and cholesterol (9:1) in chloroform was spread evenly on the indium tin oxide (ITO)-coated glass slides within the “O” ring area. The solvent was evaporated at room temperature, and the slides were dried overnight under vacuum. Then, ITO slides were assembled in the Vesi Prep Pro and filled with 275 µL of sorbitol solution (1 M). A sinusoidal AC field of 3 V and 5 Hz was applied for 2 h at 25 °C temperature. The GUV solution prepared was collected and stored at 4 °C.

### Conductance measurements in vesicles

Conductance measurements were performed using the Port-a-Patch setup (Nanion, Munich, Germany). First, a borosilicate glass chip (NPC chip, Nanion, Germany) with 3–5 mΩ was loaded with symmetrical working buffers containing 1 M MCl (M = Li^+^, Na^+^, K^+^, Rb^+^, NH_4_^+^, Li^+^, and Cs^+^), and 10 mM HEPES (pH 7.0) in both *cis* and *trans* compartments and Ag/AgCl electrodes were placed on both sides of the NPC chip. Next, bilayer membrane with >1 Giga Ohm resistance was constructed across the micrometer-sized aperture in the NPC chip by adding GUV suspension and applying a small negative pressure (−20 mbar). **MG/***h-TELO* (60 μM) was added to the *cis* side of the chamber. Current traces were recorded using an HEKA EPC 10 patch clamp amplifier with a built-in 1 kHz 4 pole Bessel low-pass filter and a Digidata 1322 A digitizer. The I–V curve was generated using a voltage ramp from −60 mV to +60 mV. For ion-transport inhibition, TO (4 equiv.) and cytosine (3 equiv. of total **MG** concentration) to the *cis* side after a stable ion transport by **MG/***h-TELO* was established. Data analysis was performed using Clampfit 10.6 software.

### Cell culture and voltage–clamp measurements in cells

CHO (ATCC) and K-562 (National Centre for Cell Science (NCCS), Pune, India) cells were cultured in 25 cm^2^ flasks in DMEM (high glucose) and 1:1 F12K/DMEM supplemented with 10% FBS (GIBCO, heat inactivated, US origin, catalogue number 10082147) at 37 °C, 5% CO_2_. Cells were harvested, and the culture media was substituted with external solution (10 mM HEPES, pH 7.4) with 150 mM of KCl. For these experiments, we have used 3–5 mΩ NPC chips to form bilayer across its micrometer-sized aperture. Next similar to vesicle experiments, the **MG/***h-TELO* (60 μM) was added to the *cis* side of the chip, and the data were recorded. For each type of cells, we performed 20 replica experiments and analyzed the data. The data acquisition and analysis were carried out similar to vesicle experiments.

### Statistics

Results are expressed as means ± SD of *n* number of independent experiments.

### Reporting summary

Further information on research design is available in the Nature Research Reporting Summary linked to this Article.

## Supplementary information

Supplementary Information

Supplementary Movie 1

Reporting Summary

Description of Additional Supplementary Files

## Data Availability

Data supporting the findings of this paper are available from the corresponding author upon reasonable request. A Reporting Summary for this Article is available as a Supplementary Information file. The source data underlying Figs. 1–8 and Supplementary Figs. 1, 2, 4, 7, 20–22, and 25 are provided as a Source Data file. Data available on request from the authors.

## Source data

Source Data
