# Supplementary material for: Ionophore constructed from non-covalent assembly of a G-quadruplex and liponucleoside transports K+-ion across biological membranes
Source: Nat Commun. 2020 Jan 24;11:469. doi: 10.1038/s41467-019-13834-7 (PMC6981123; doi:10.1038/s41467-019-13834-7)
Supplement: Supplementary file 1 — Supplementary Information [file 41467_2019_13834_MOESM1_ESM.pdf]

SUPPLEMENTARY INFORMATION

Ionophore Constructed from Non-covalent Assembly of a G-quadruplex and Liponucleoside Transports K^+-ion Across Biological Membranes

Manish Debnath, Sandipan Chakraborty, Y. Pavan Kumar, Ritapa Chaudhuri, Biman Jana, Jyotirmayee Dash*

School of Chemical Sciences, Indian Association for the Cultivation of Science, Jadavpur, Kolkata-700032, India; email:ocjd@iacs.res.in

Supplementary Methods

General information

The experiments were carried out under an inert atmosphere unless otherwise stated. Solvents were purified by standard procedures. All starting materials were obtained from commercial suppliers and used as received. Products were purified by flash chromatography on silica gel (100-200 mesh, Merck). Unless otherwise stated, yields refer to analytical pure samples. ^{1}H and ^{13}C NMR spectra for monitoring the synthesis of the ligands were recorded on either Bruker AVANCE 500 (500MHz and 125 MHz), or Bruker AVANCE 400 (400 MHz and 100 MHz) instruments. NMR spectra were recorded in deuterated solvents as detailed at ambient probe temperature (300 K). Chemical shifts are reported in parts per million (ppm) and are referenced to the residual solvent peak (^{1}H: $\delta(CDCl_3)$ = 7.26 ppm, $\delta((CD_3)_2SO)$ = 2.50 ppm and ^{13}C: $\delta(CDCl_3)$ = 77.16 ppm, $\delta(CD_3)_2SO)$ = 39.52 ppm). Signals are quoted as δ values in ppm and coupling constants (*J*) are reported in Hertz. Infrared (FTIR) spectra (ν_{max}) are recorded on a Perkin Elmer spectrophotometer using KBr disk techniques for solid compounds and as a *thin film* for liquid samples and are reported in cm^{-1}. HRMS analysis was performed with Micromass Q-Tof micro (Water Corporation) instruments by +ve mode electrospray ionization. The general chemicals and DNA sequences required for biophysical analysis were purchased from Sigma-Aldrich. The DNA sequences of highest purity were purchased for best results. The lipid and cholesterol was purchased from Sigma-Aldrich. The voltage clamp studies were performed using Nanion port-a-patch (Germany) system.

Synthesis of guanosine derivative (MG)

The biocompatible guanosine derivative **MG** was synthesized using a 'one-pot' modular synthetic approach based on Cu (I)-catalyzed azide and alkyne cycloaddition.[1] Initially, the azide unit was incorporated into the natural nucleoside guanosine **1** to synthesize clickable guanosine building block **2** using two to three steps. The lipophilic monoalkyne linker **5** was prepared by amide coupling of stearic acid **3** with propargyl amine **4** using HBTU and DIPEA in dry DMF in 76% yield.

2', 3'-Isopropylideneguanosine: Perchloric acid (70%, 2.44 mL, 28.4mmol) was added to a stirring suspension of guanosine (6.0 g, 21.18 mmol) in 360 mL dry acetone. The reaction mixture was stirred for 1.5 h at rt. The reaction was quenched with drop wise addition of ammonium hydroxide (4.02 mL, 29.7 mmol) at 0 °C. The solid obtained was filtered out and dried under vacuum to afford 2', 3'-isopropylideneguanosine as a white solid (5.82 g , 85%); ^{1}H NMR (400 MHz, DMSO-d_6): 10.66 (s_{br}, 1H), 7.91 (s, 1H), 6.50 (s_{br}, 2H), 5.91 (d, *J* = 2.7 Hz, 1H), 5.17 (dd, *J* = 7.81, 3.45 Hz, 1H), 5.04 (t, *J* = 6.7 Hz, 1H), 4.97-4.94 (m, 1H), 4.11-4.09 (m, 1H), 3.52-3.35 (m, 2H), 1.50 (s, 3H), 1.30 (s, 3H); ^{13}C NMR (100 MHz, DMSO-d_6): 157.1, 153.9, 150.5, 135.9, 116.9, 113.0, 88.4, 86.6, 83.5, 81.1, 61.6, 27.0, 25.2.

5'-O-Chlorobutyryl-2',3'-O-isopropylidene guanosine: To a suspension of 2', 3'-isopropylideneguanosine (2.0 g, 6.19mmol) in dry pyridine (50 mL) was added catalytic amount of DMAP (75 mg, 0.62 mmol) and a solution of 4-chlorobutyryl chloride (1.59 mL, 14.24 mmol) in dry CH_2Cl_2 (30 mL). The reaction mixture was stirred at rt under an argon atmosphere for 16 h. The reaction was quenched with drop wise addition of methanol (2 mL) and concentrated under reduced pressure. Ice water was added to this mixture and stirred for 30 min. The obtained solid product was filtered and washed with water followed by 40% ethyl acetate in hexane and dried under vacuum to afford 5'-O-chlorobutyryl-2',3'-O-isopropylidene guanosine as a brown solid (1.75 g, 66%): R_f = 0.4 (10% MeOH in CH_2Cl_2);^{1}H NMR (400 MHz, DMSO-d_6): 10.70 (s_{br}, 1H), 7.84 (s, 1H), 6.53 (s_{br}, 2H), 6.00 (d, *J* = 1.9 Hz ,1H), 5.26-5.23 (m, 1H), 5.14-5.11(m, 1H), 4.28-4.23 (m, 2H), 4.16-4.10 (m, 1H), 3.61 (t, *J* = 6.6 Hz, 3H), 2.45-2.40 (m, 2H), 1.96-1.88 (m, 2H), 1.50 (s, 3H), 1.31 (s, 3H); ^{13}C NMR (75 MHz, DMSO-d_6): 171.9,156.7, 153.7, 150.5, 136.2, 117.0, 113.3, 88.3, 84.2, 83.7, 81.1, 64.1, 44.4, 30.6, 27.4, 27.0, 25.3; IR (KBr): 3425, 3308, 3167, 1699, 1634, 1601, 1481, 1377, 1213, 1178, 1103, 866; HRMS (ESI) calcd for $C_{17}H_{23}ClN_5O_6$ [M+H]$^{+}$: 428.1331; Found 428.1332.

5'-O-Azidobutyryl-2',3'-O-isopropylidene guanosine 2: To a stirring suspension of 5'-O-chlorobutyryl-2',3'-O-isopropylidene guanosine (2.0 g, 4.7 mmol) in DMF (20 mL) was added sodium azide (1.52 g, 23.4 mmol). The reaction mixture was stirred at 100 °C for 24 h. The reaction mixture was cooled to rt and concentrated to minimum amount of solvent (5 mL). Ice water was added to this mixture and stirred for 30 min. The mixture was filtered and washed with water followed by 40% ethyl acetate in hexane and dried under vacuum to yield 5'-O-azidobutyryl-2',3'-O-isopropylidene guanosine **2** as a brown solid (1.80 g, 79%). R_f = 0.4 (10% MeOH in CH_2Cl_2); ^{1}H NMR (400 MHz, DMSO-d_6): 10.80 (s_{br}, 1H), 7.84 (s, 1H), 6.59 (s_{br}, 2H), 6.00 (d, J = 1.9 Hz,1H), 5.27-5.23 (m, 1H), 5.15-5.11 (m, 1H), 4.30-4.20 (m, 2H), 4.17-4.10 (m, 1H), 3.31 (m, 2H, merged with water peak), 2.40-2.30 (m, 2H), 1.80-1.70 (m, 2H), 1.51 (s, 3H), 1.32 (s, 3H); ^{13}C NMR (125 MHz, DMSO-d_6): 172.0, 157.3, 154.0, 150.6, 136.1, 117.0, 113.3, 88.3, 84.1, 83.6, 81.1, 64.1, 49.9, 30.4, 27.0, 25.3, 23.7; IR(KBr): 3398, 3165, 2100, 1749, 1691, 1603, 1387, 1178, 1070; HRMS (ESI) calcd for $C_{17}H_{23}N_8O_6[M+H]^+$: 435.1735; Found 435.1736.

Synthesis of *N*-(prop-2-yn-1-yl) stearamide 5: To a solution of stearic acid **3** (350 mg, 1.2 mmol) in dry DMF (8 mL), propargyl amine **4** (0.24 mL, 3.7 mmol), DIPEA (1.1 mL, 6.2 mmol) and HBTU (1.4 g, 3.7 mmol) were added and the mixture stirred at room temperature for 16 h. The reaction mixture was then diluted with cold water and stirred for 1 h. The solid formed was filtered, washed with cold water and dried under vacuum to yield *N*-(prop-2-yn-1-yl)stearamide **5** as a white solid (301 mg, 76%). ^{1}H NMR (500 MHz, $CDCl_3$) δ 5.57 (s, 1H), 4.06-4.04 (m, 2H), 2.23 (t, J = 2.6 Hz, 1H), 2.19 (t, J = 7.6 Hz, 2H), 1.66-1.61 (m, 2H), 1.33-1.27 (m, 28H), 0.88 (t, J = 6.7, 3H); ^{13}C NMR (100 MHz, $CDCl_3$) δ 172.8, 79.8, 71.7, 36.7, 32.1, 29.9, 29.8, 29.7, 29.6, 29.5, 29.5, 29.4, 29.3, 25.7, 22.8, 14.3.; IR (KBr, cm^{-1}) 3294, 3072, 2957, 2918, 2849, 1639, 1554, 1462, 1381, 690, 633, 571; HRMS (ESI) calcd for $C_{21}H_{40}NO^+$ $[M+H]^+$: 322.3104; Found 322.3104.

Synthesis of MG by Cu-(I) catalyzed alkyne azide cycloaddition: To a suspension of alkyne **5** (130 mg, 1.0 equiv.) and guanosine azide **2** (193 mg, 1.1 equiv. per alkyne group) in 4 mL *t*BuOH:H_2O (1:1) was added sodium ascorbate (0.2 equiv.) followed by $CuSO_4.5H_2O$ (0.1 equiv.). The reaction mixture was stirred for 24 h at rt. The reaction mixture was concentrated and the residue was purified by flash chromatography to provide **MG**. Flash chromatography over silica gel (eluent 7% MeOH in CH_2Cl_2) yielded **MG** as an off white solid (159 mg, 52% yield). R_f = 0.4 (8% MeOH-CH_2Cl_2); ^{1}H NMR (500 MHz, DMSO-d_6) δ 10.71(s_{br}, 1H), 8.23 (t, J = 5.7 Hz, 1H), 7.87 (s, 1H), 7.84 (s, 1H), 6.54 (s_{br}, 2H), 6.01 (d, J = 2.2 Hz, 1H), 5.25 (d, J = 6.1 Hz, 1H), 5.15-5.12 (m, 1H), 4.32 (t, J = 7.2 Hz, 2H), 4.27-4.22

(m, 4H), 4.15-4.10 (m, 1H), 2.31-2.26 (m, 2H), 2.06 (t, *J* = 7.6 Hz, 2H), 2.03-1.96 (m, 2H), 1.51 (s, 3H), 1.49-1.45 (m, 2H), 1.31 (s, 3H), 1.26-1.20 (m, 28H), 0.85 (t, *J* = 6.1 Hz, 3H); ^{13}C NMR (125 MHz, DMSO-d_6) δ 172.0, 171.7, 156.7, 153.6, 150.5, 145.0, 136.1, 122.7, 117.0, 113.3, 88.3, 84.1, 83.6, 81.0, 64.1, 48.3, 35.1, 34.1, 31.2, 30.1, 29.0, 28.9, 28.8, 28.7, 28.6, 27.0, 25.3, 25.2, 25.1, 22.0, 13.9; IR (KBr): 3443, 3306, 2918, 2851, 1737, 1710, 1640, 1600, 1539, 1487, 1381, 1213, 1067, 777; HRMS (ESI) calcd for $C_{38}H_{61}N_9NaO_7^+$: $[M+Na]^+$: 778.4586; Found 778.4590.

NMR spectra of all compounds

^{1}H NMR of 1

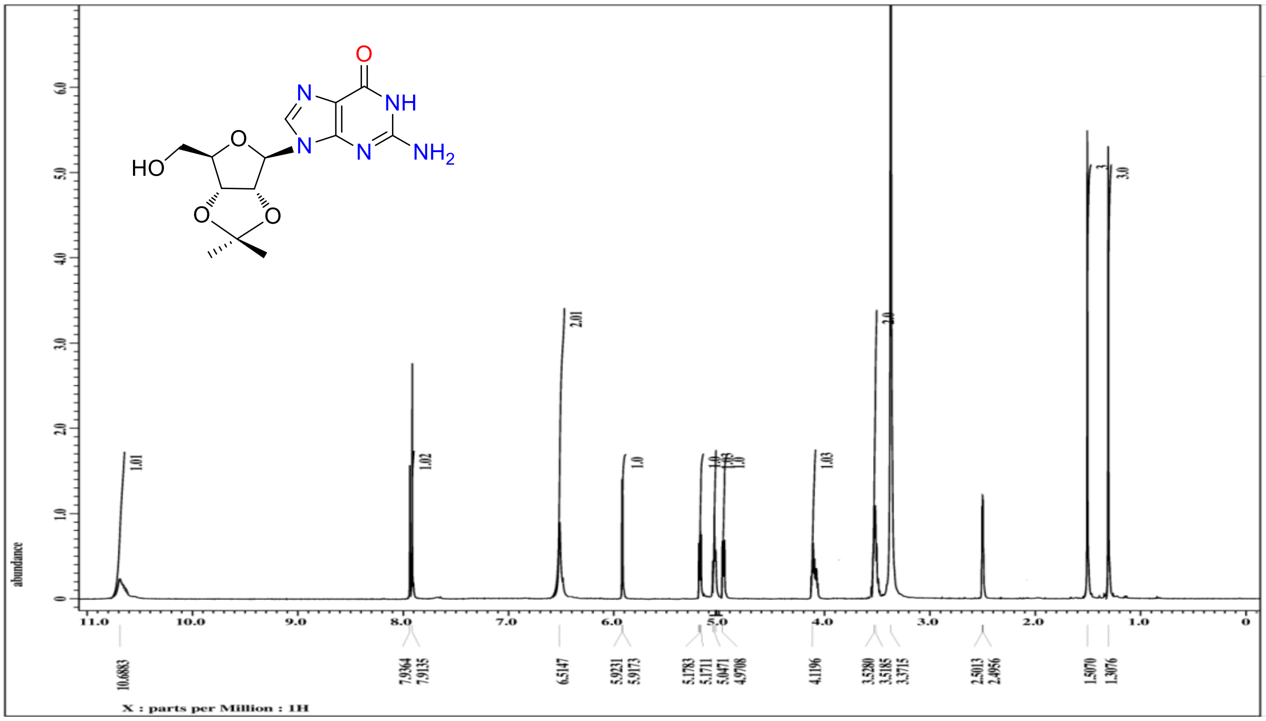

^{13}C NMR of 1

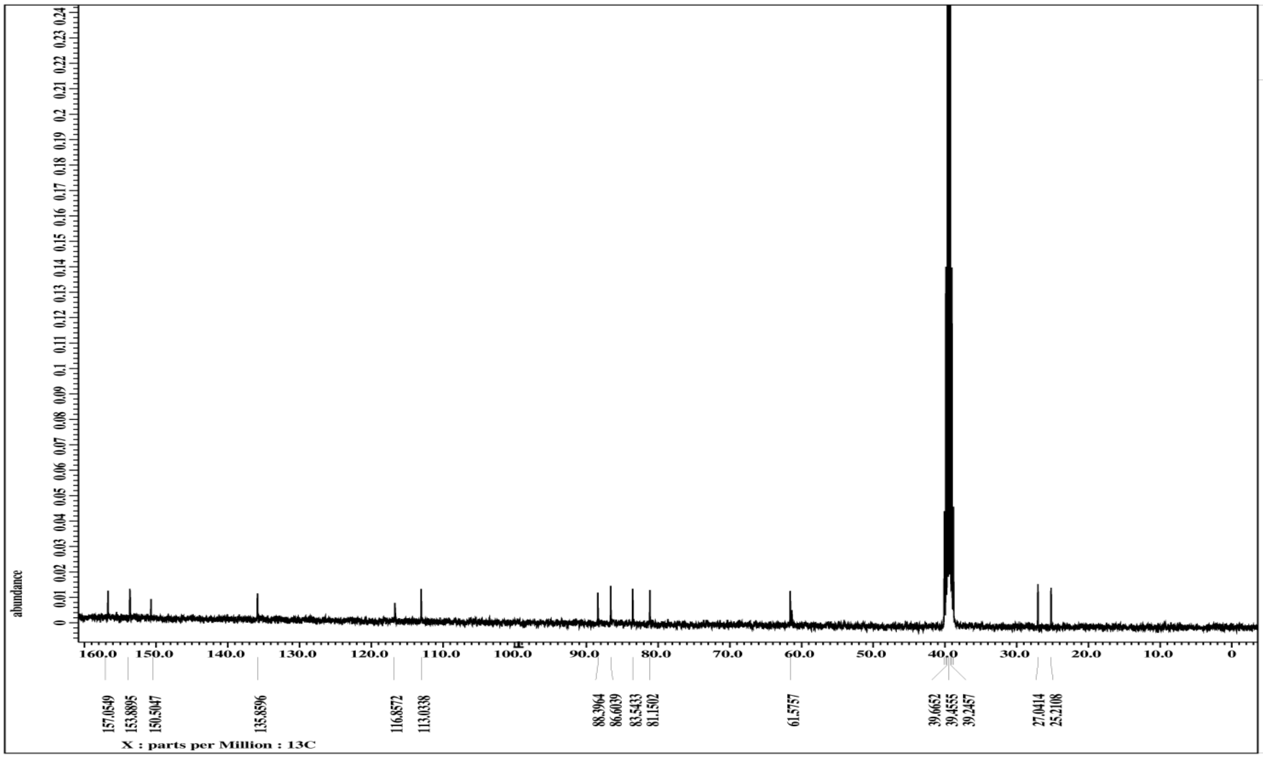

^{1}H NMR of 5'-O-chlorobutyryl-2',3'-O-isopropylidene guanosine

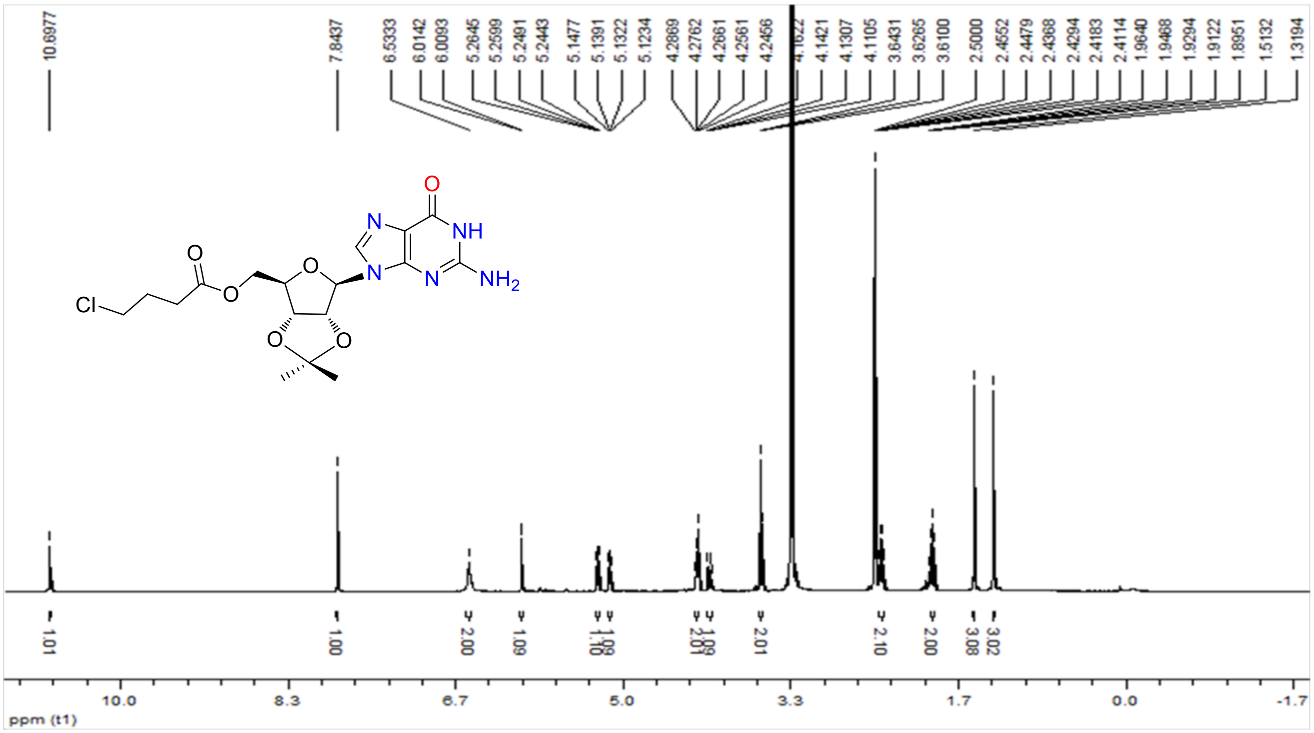

^{13}C NMR of 5'-O-Chlorobutyryl-2',3'-O-isopropylidene guanosine

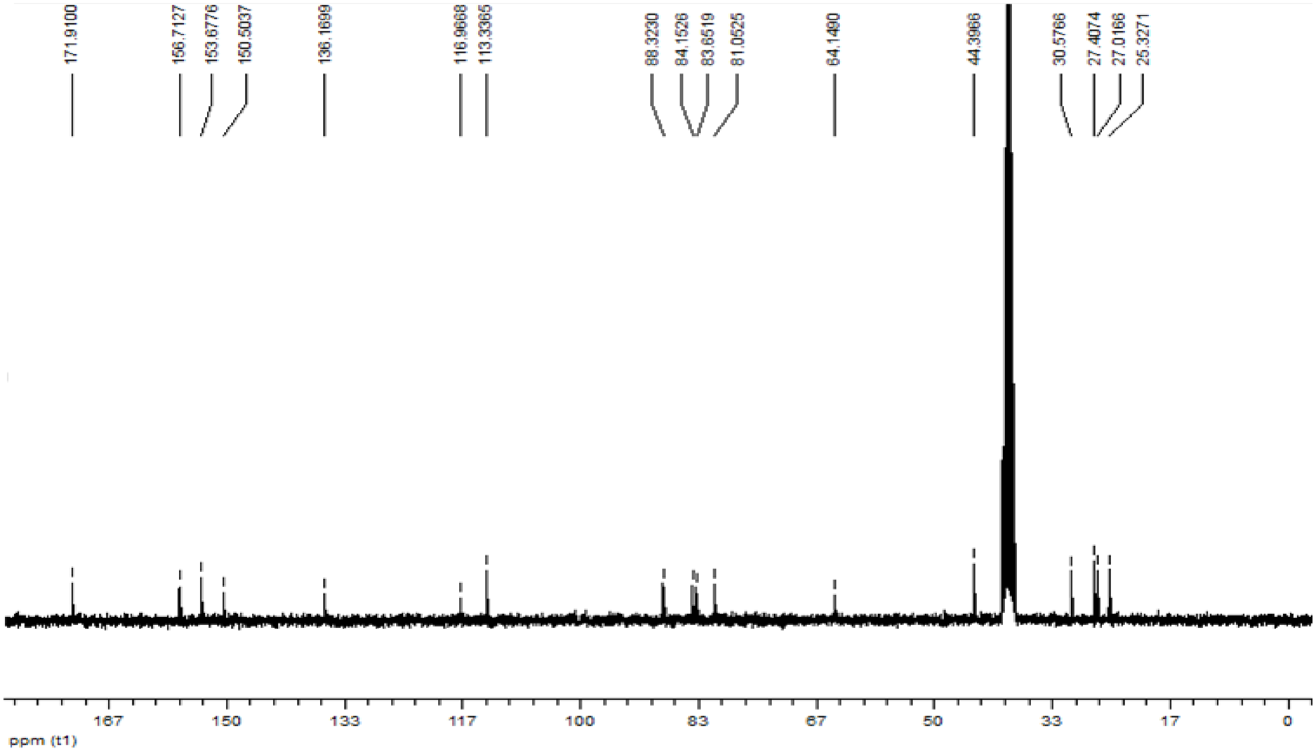

^{1}H NMR of 2

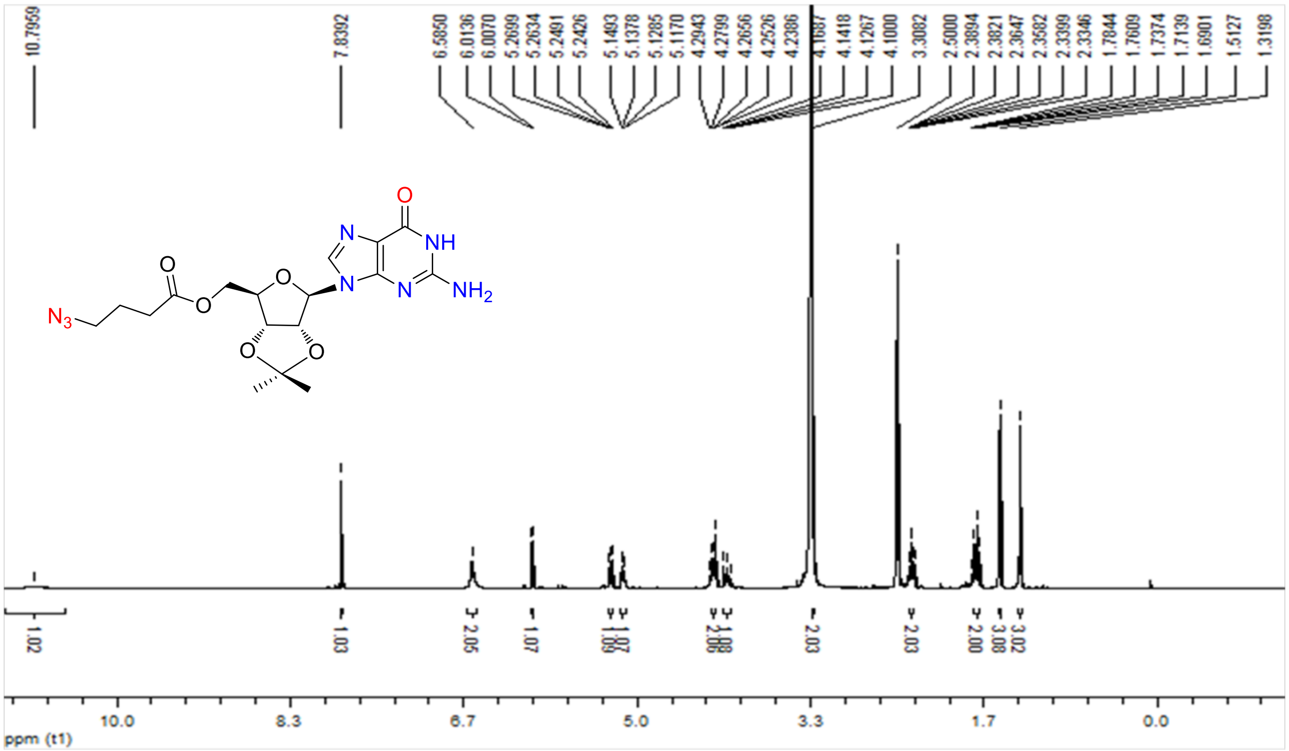

^{13}C NMR of 2

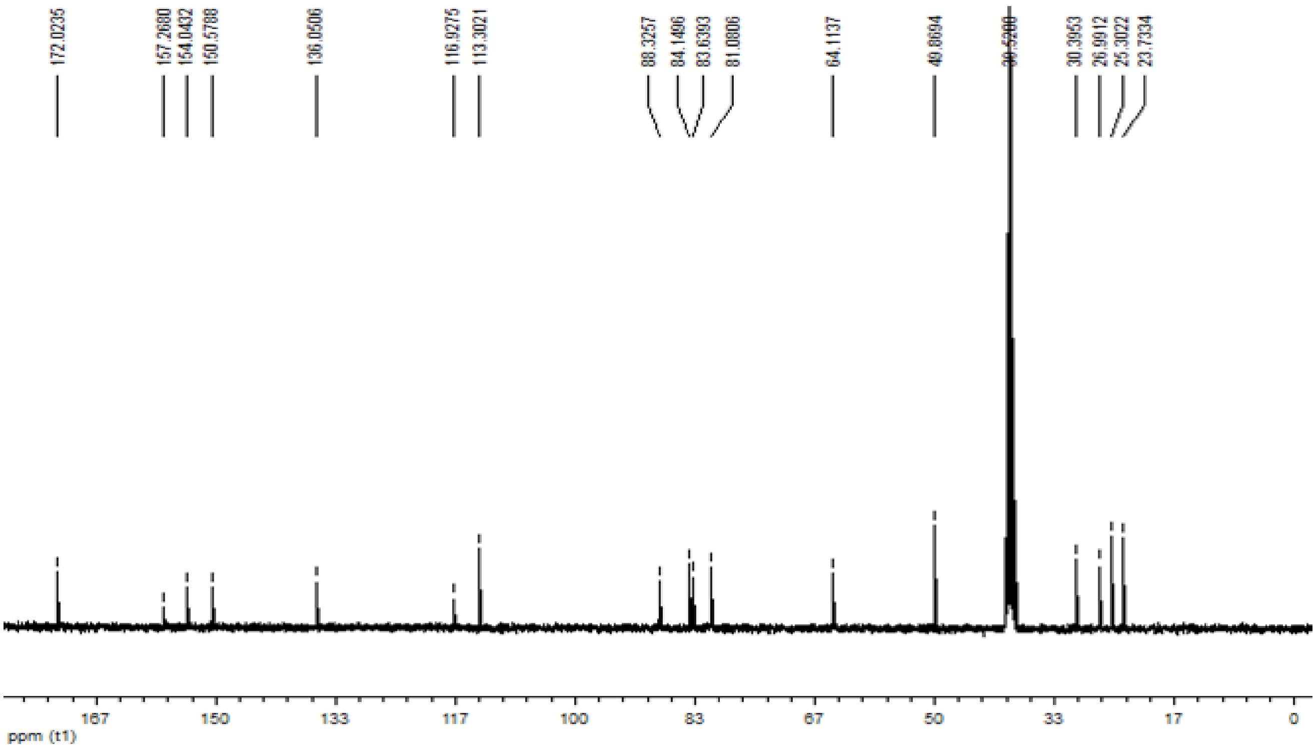

^{1}H NMR of 5

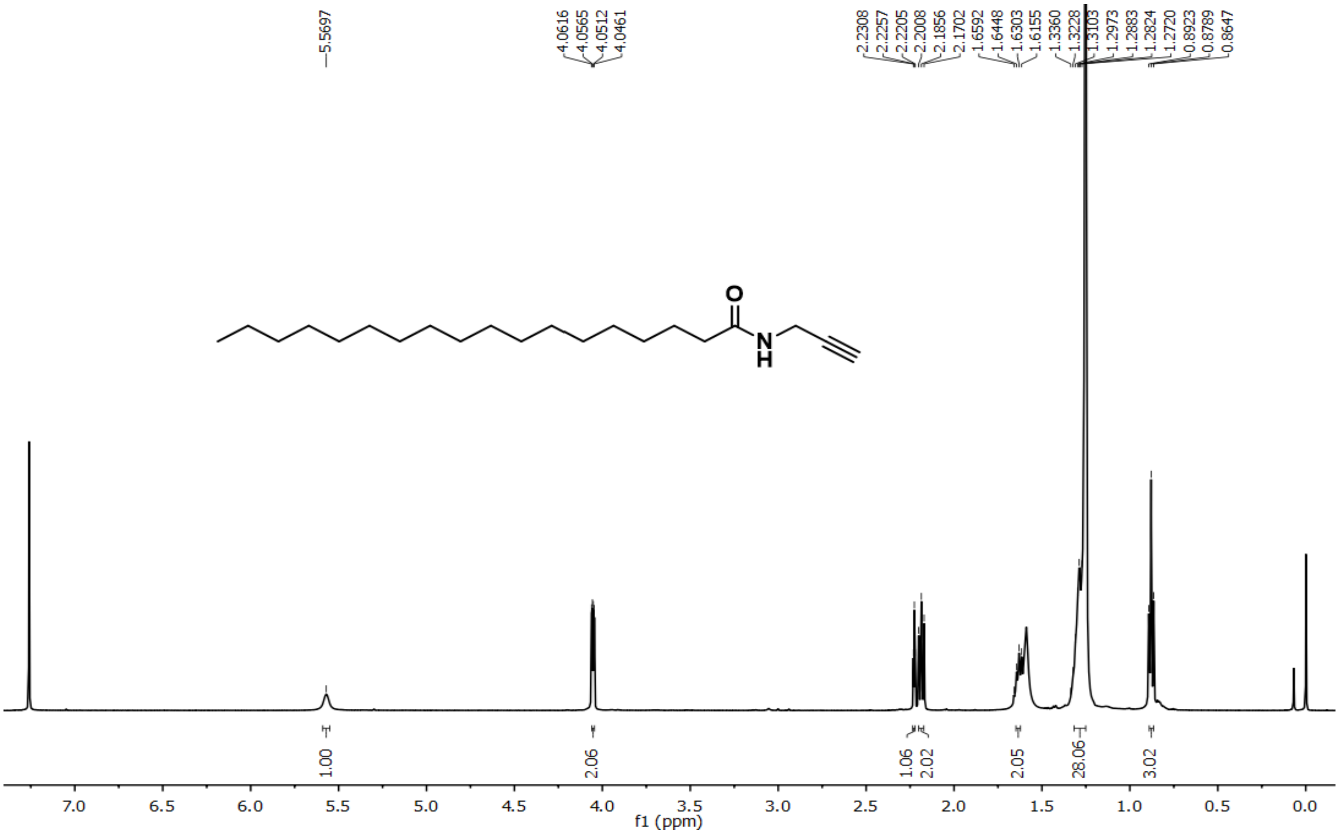

^{13}C NMR of 5

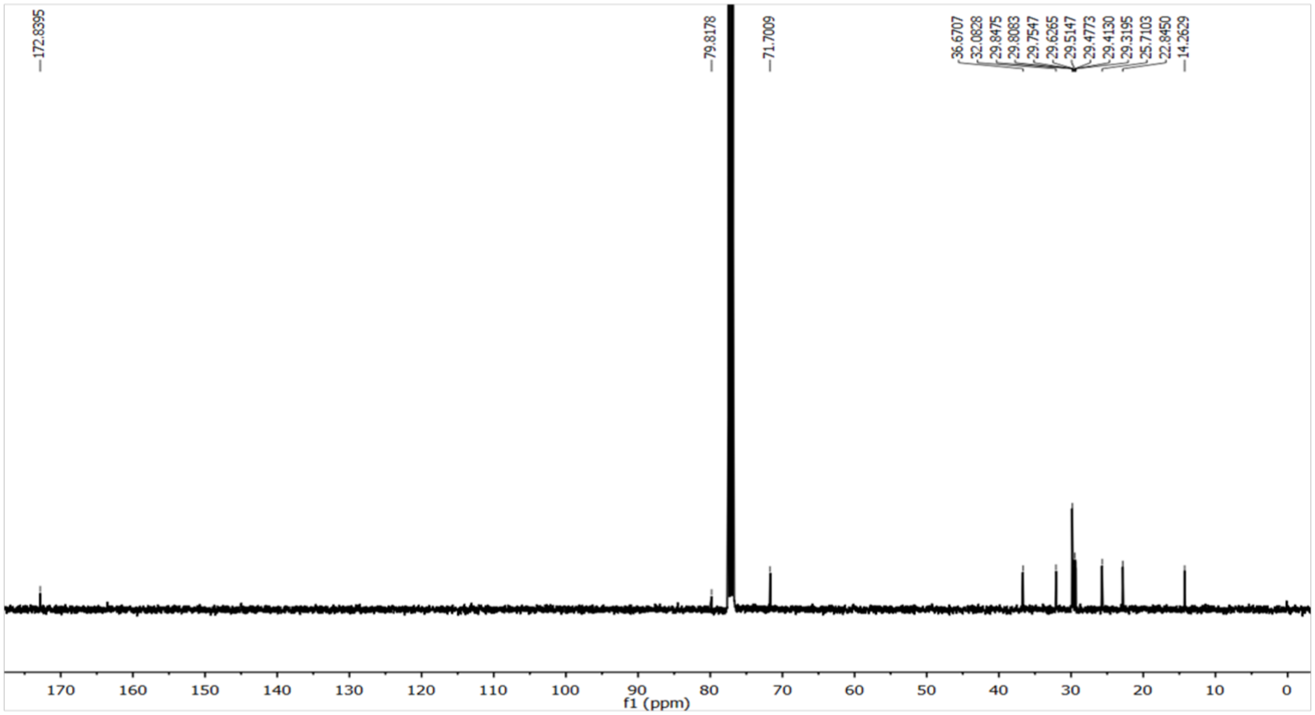

^{1}H NMR of MG

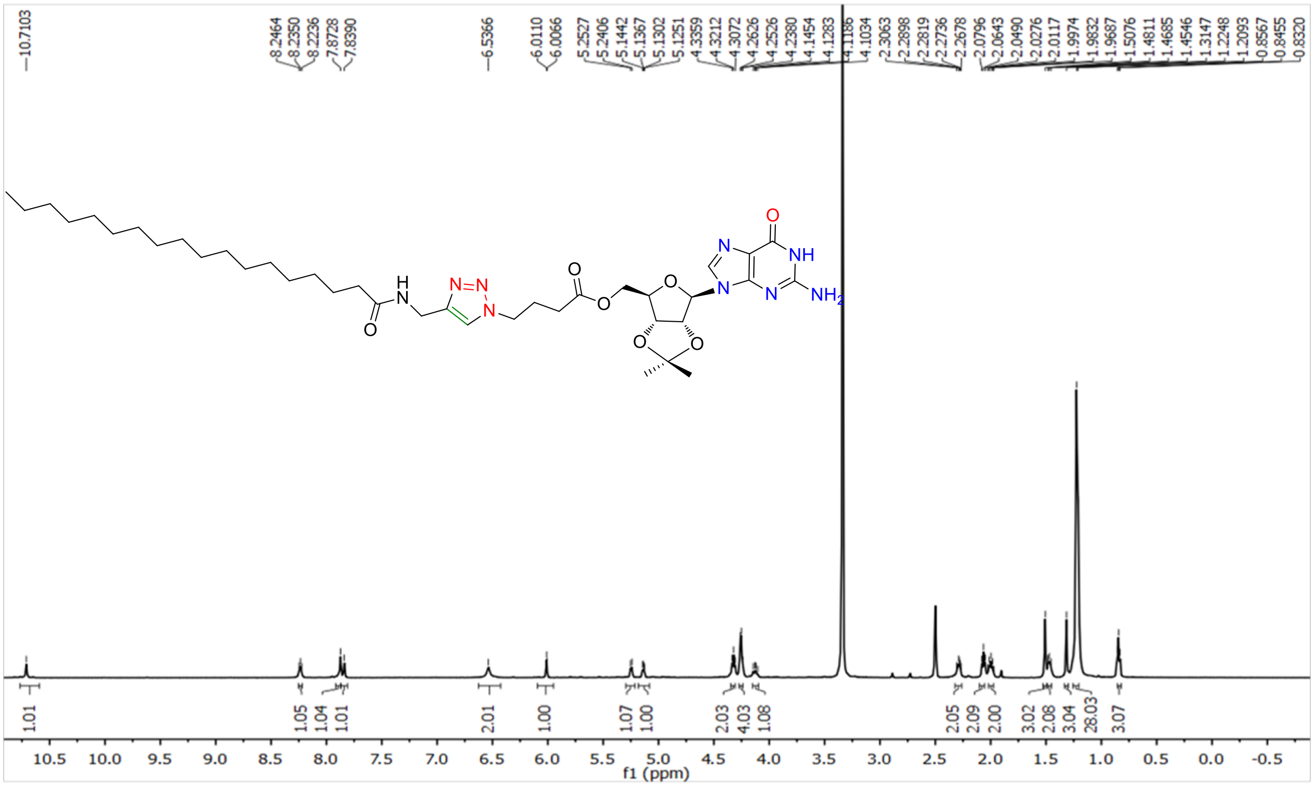

^{13}C NMR of MG

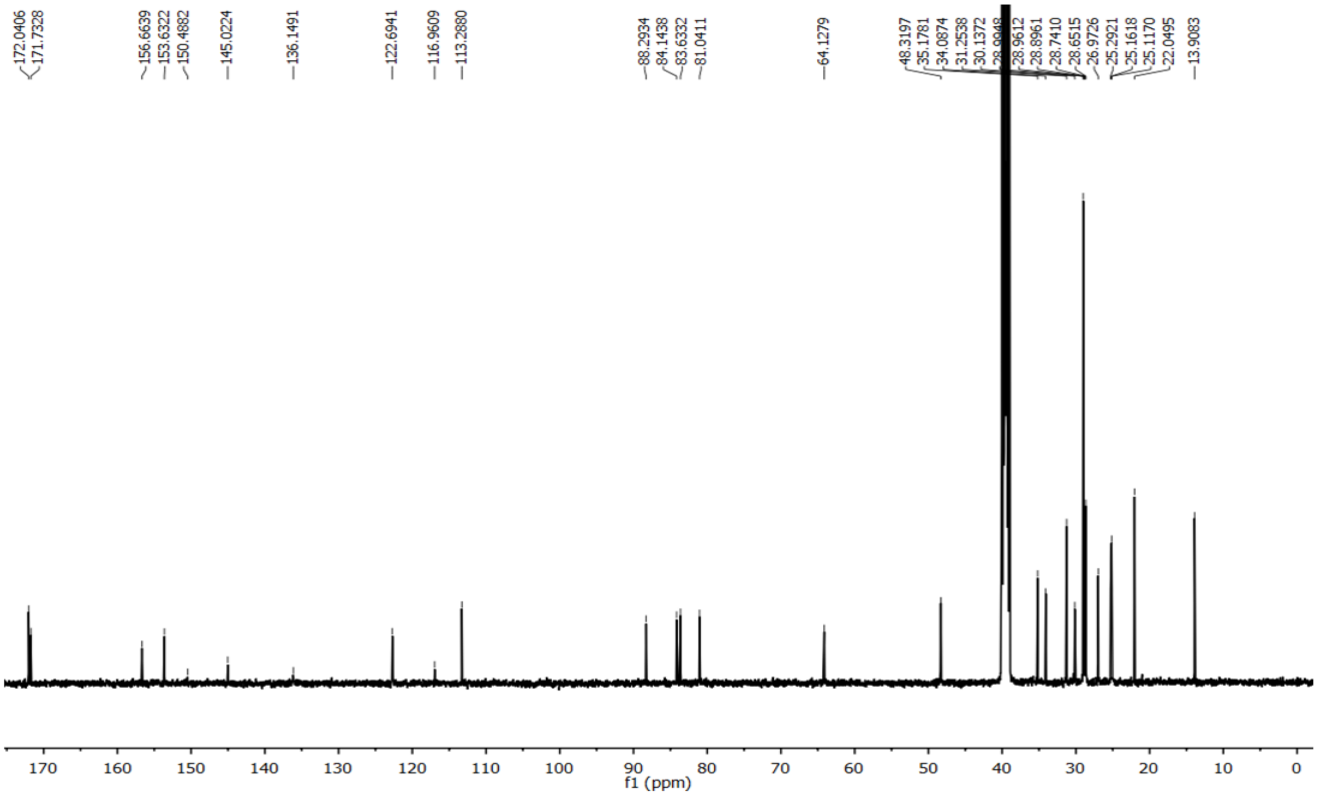

FRET melting analysis

Stock solution of **MG** (200 µM) was prepared in MQ water (pH 7.4). Dual labeled parallel-type *h-TELO* G-quadruplex forming sequence was diluted in 10 mM HEPES, 100 mM KCl buffer (pH 7.4) and annealed at a concentration of 1 µM by heating at 95 ºC for 5 min followed by cooling to room temperature. The 96-well plates were prepared by aliquoting 50 µL of the annealed DNA into each well, followed by 50 µL of **MG** at different concentrations (0.0, 0.5, 1.0, 1.5, 2.5, 3.5 µM). For melting analysis in different salt environments, five aliquots of *h-TELO* G-quadruplex forming sequence were prepared by diluting in 10 mM HEPES (pH 7.4) containing 100 mM of LiCl, NaCl, KCl, RbCl and CsCl, respectively and these solutions were annealed at a concentration of 500 nM by heating at 95 ºC for 5 min followed by cooling to room temperature. The 96-well plates were similarly prepared by aliquoting 50 µL of the annealed DNA into each well, followed by 50 µL of **MG** at a final concentration of 1.5 µM. Measurements were made in triplicate with an excitation wavelength of 483 nm and a detection wavelength of 533 nm using a LightCycler® 480-II System RT-PCR machine (Roche). Final analysis of the data was carried out using Origin Pro 8 data analysis**.**

Sequence used in this study,

h-TELO: 5'-FAM-d($AG_3TTAG_3TTAG_3TTAG_3$)-TAMRA-3'

Fluorescence binding titration of MG with *h-TELO*

The fluorescence experiments were carried out using a Horiba JobinYvon Fluorolog-3 instrument at 25 °C in a thermostated cell holder using quartz cuvette of 1 cm path-length. For fluorescence titration of **MG** with *h-TELO* G-quadruplex, 200 µM of *h-TELO* was pre-annealed in filtered and degassed 10 mM HEPES, 100 mM KCl buffer (pH 7.4) by heating at 95 °C and subsequent cooling to room temperature. To a solution of **MG** (10 µM), the pre-annealed *h-TELO* was added in a stepwise manner (0, 0.5, 1.0, 1.5, 2.0, 2.5, 3.0, 3.5, 4.0, 4.5, 5.0, 5.5, 6.0 µM) and subsequently the fluorescence emission was recorded after a 2 min equilibration period. **MG** was excited at 265 nm and the fluorescence emission spectra were recorded from 300 nm to 540 nm using 4 nm bandpass filters. The spectral data represents the mean of three scans and the final analysis of the data was carried out using Origin 8.

Sequence used in this study:

h-TELO: 5'-d($AG_3TTAG_3TTAG_3TTAG_3$)-3'

The recorded spectral data was used to determine the dissociation constant of the ligands for quadruplexes using the Hill-1 formula (1):[2]

$$F = F_0 + \frac{(F_{max} - F_0)[DNA]}{K_{\mathrm{d}} + [DNA]} \; .... (1)$$

F is the fluorescence intensity, F_{max} is the maximum fluorescence intensity, F_0 is the fluorescence intensity in the absence of DNA and K_{d} is the dissociation constant.

Job's plot analysis

Binding stoichiometries were measured by continuous variation binding analysis by the method according to Job.[3] Fluorescence spectra were recorded on a Horiba JobinYvonFluorolog 3 instrument at 25 °C in a thermostated cell holder using quartz cuvette of 2 mm path-length. **MG** and *h-TELO* were varied for a fixed and constant summed concentration of 10 µM. The mole fractions of **MG** ranging from (0, 0.1, 0.2, 0.3, 0.4, 0.5, 0.6, 0.7, 0.8, 0.9, 1.0) in 10 mM HEPES, 100 mM KCl buffer (pH 7.4), were added to the *h-TELO* (mole fraction: 1.0, 0.9, 0.8, 0.7, 0.6, 0.5, 0.4, 0.3, 0.2, 0.1, 0). Excitation wavelength was 265 nm and emission wavelengths from 300 to 540 nm. Each reading is a mean of three scans.

Isothermal titration calorimetry (ITC)

ITC measurements were carried out in MicroCal PEAQ-ITC microcalorimeter. Before experiment, the solutions were thoroughly degassed. The reference cell was filled with the degassed 10 mM HEPES, 100 mM KCl (pH 7.4) buffers. The preformed *h-TELO* quadruplex (5 µM) in buffer was kept in the sample cell and the ligand **MG** (100 µM) was filled in the syringe of volume 40 µL in the same buffer. Ligand solution was added sequentially, mixing was carried out by stirring the syringe at a speed of 1000 rpm at 298 K. 38 injections with duration of 2 s and 150 s of spacing between two injections were set up. Blank titrations were conducted by injecting the ligand into the cell containing only buffer under the identical conditions. The heat generated due to interaction was determined by subtracting the blank heat from that for the ligand-*h-TELO* titration. Finally, binding enthalpies were obtained by fitting the corrected data to an appropriate binding model.

Fluorescence Microscopy for membrane localization

Preparation of Giant Unilamellar Vesicles (GUVs) for fluorescence microscopy

The GUVs were prepared using electroformation technique (Vesi Prep Pro, Nanion, Germany). 10 µL of a 10 mM solution of DphPC (1,2-diphytanoyl-sn-glycero-3-phosphocholine) and cholesterol (9:1) in chloroform was spread evenly on the indium tin oxide (ITO) coated glass slides within the "O" ring area. The solvent was evaporated at room temperature and the slides were dried overnight under vacuum. Then, ITO slides were assembled in the Vesi Prep Pro and filled with 275 µL of sorbitol solution (1 M). A sinusoidal AC field of 3 V and 5 Hz was applied for 2 h at 25 °C temperature. The GUV solution prepared was collected and stored at 4 °C.

Fluorescence Microscopy

GUVs were suspended in 10 mM HEPES, 100 mM KCl, pH 6.4 buffer and incubated with **MG-**bound *h-TELO* (20 µM) for 5 minutes. After incubation, the mixture was placed on the glass slide and cover slip was placed over it. The slide was viewed under Olympus IX 81 Microscope. For control slides, GUVs were incubated with either *h-TELO* or **MG.** At least 4 fields per slide and three independent sets were examined. The images obtained were processed using ImageJ software.

LUV preparation for HPTS experiments

Large unilamellar vesicles (LUVs) were formed using a 20 µL 9:1 mixture of 10 mM DphPC phospholipid and cholesterol in chloroform. The solvent was slowly removed by evaporation under vacuum at room temperature and dried overnight under high vacuum. The resulting thin film was hydrated with 500 µL buffer (10 mM HEPES, 100 mM NaCl or KCl, pH 6.4) containing 10 µM HPTS (8-hydroxypyrene-1,3,6-trisulfonic acid trisodium salt). Next, the suspension was subjected to six freeze-thaw cycles (liquid nitrogen/water at room temperature) during hydration. The resulting white suspension was then extruded 19 times through a 100 nm polycarbonate membrane to obtain large unilamellar vesicles (LUVs) with an average diameter of ~ 100 nm. The LUVs suspension was separated from extra-vesicular HPTS dye by using size exclusion chromatography (Econo-Pac 10DG column, Bio-rad; mobile phase: 10 mM HEPES, 100 mM NaCl or KCl, pH 6.4) and diluted with mobile phase to desired working concentration.

Cation transport experiments

In a clean and dry fluorescence cuvette, 5 µL of stock HPTS containing vesicle solution was suspended in 495 µL of the corresponding buffer (10 mM HEPES, 100 mM KCl, NaCl, LiCl, NH_4Cl and CsCl; pH 6.4). Rb salts somehow interfered with the assay and hence excluded. The fluorescence of HPTS at 510 nm was monitored upon excitation at 460 nm in a time dependent manner. For pH gradient mediated transport experiments, different concentrations of **MG**/*h-TELO* (0, 10, 20, 30, 40, 50, 60 µM) was mixed initially (at t = 0 s) with vesicles. At t = 50 s, 7.25 µL of aqueous NaOH (0.5 M) was added, resulting in a pH increase by one unit in the extravesicular solution. Finally, at 350 s, vesicles were lysed with detergent (10 µL of 5% aqueous Triton X-100) resulting in destruction of pH gradient. For passive diffusion studies, 5 µL of stock HPTS containing vesicle solution was similarly suspended in 495 µL of the corresponding buffer (10 mM HEPES, 100 mM 100 mM KCl, NaCl, LiCl and CsCl, pH 6.4) as in case of pH gradient studies. At t = 50 s, different concentrations of **MG**/*h-TELO* (0, 10, 20, 30, 40, 50, 60 µM) was added and the fluorescence of HPTS at 510 nm was monitored till 350 s.

Dynamic light scattering. The average hydrodynamic radius (R_h) of **MG**/*h-TELO* embedded in vesicles was evaluated by DLS using a particle size analyzer (Zetasizer Nano-ZS, Malvern Instruments Ltd.). The sample was prepared by gel filtration of **MG**/*h-TELO* embedded LUVs suspension to a final concentration of 20 µM **MG**/*h-TELO* in 40 µM of LUV suspension in 10 mM HEPES, 100 mM KCl, pH 7.4. Measurements were recorded at room temperature (25 °C) with a scattering angle of 90 °.

Preparation of Lucigenin filled LUVs

The LUVs were formed in a small round bottom flask using a 20 µL 9:1 mixture of 10 mM phospholipid and cholesterol in chloroform. The solvent was evaporated by a stream of nitrogen, followed by drying under vacuum for 4-5 h. After that 0.5 mL of 1 mM lucigenin (N,N′-dimethyl-9,9′-biacridinium dinitrate) in 200 mM $NaNO_3$ buffer, pH 7.4 was added and the mixture was hydrated for 1 h with occasional vortexing. The mixture was subjected to freeze-thaw cycle (6 times) and the resulting suspension was then extruded 19 times through a 200 nm polycarbonate membrane to obtain large unilamellar vesicles (LUVs) with an average diameter of ~ 200 nm. The extracellular lucigenin was removed from the vesicles by size exclusion column chromatography (Econo-Pac 10DG column, Bio-rad; mobile phase: 10 mM HEPES, 100 mM NaCl or KCl, pH 6.4) using 200 mM $NaNO_3$ as eluent. The obtained vesicles were diluted with 200 mM $NaNO_3$ to desired working concentration.

Determination of chloride ion selectivity by lucigenin assay

10 μL of lucigenin filled LUV solution and 490 μL of 200 mM $NaNO_3$ solution was taken in a cuvette in the fluorescence spectrometer. Here the fluorescence emission of lucigenin was monitored at λ_{em}= 535 nm (λ_{ex} = 455 nm). At t = 50 s, 6 μL of 2 N NaCl was added to the mixture to establish a salt gradient across the bilayer. At t = 200 s, **MG**/*h-TELO* (60 μM) was added and the corresponding fluorescence was monitored. For positive control, at t = 200 s, 10 μL of 5% aqueous Triton X-100 was added to lyse all vesicles for 100% chloride influx.

Membrane polarization experiments

For membrane polarization experiments, LUVs were prepared using 20 μL 9:1 mixture of 10 mM phospholipid and cholesterol in chloroform in a small round bottom flask. Then the flask was dried as described for HPTS assay and the lipid cake is hydrated in 500 μL of 10 mM HEPES, 100 mM NaCl or KCl, pH 6.4. In a fluorescence cuvette, 25 μL of LUV solution was suspended in 475 μL 10 mM HEPES, pH 6.4 containing the corresponding salt 100 mM NaCl or KCl. Next, safranin O was added at a final concentration of 60 nM. The fluorescence was monitored at an excitation of 522 nm and emission of 581 nm. At t = 50 s, **MG**/*h-TELO* (60 μM) was added to the mixture and the fluorescence emission was monitored for 600 sec.

Preparation of Giant Unilamellar Vesicles (GUVs) for patch clamp experiments

For patch clamp experiments, we prepared GUVs using electroformation technique (Vesi Prep Pro, Nanion, Germany). 10 μL of a 10 mM solution of DphPC and cholesterol (9:1) in chloroform was spread evenly on the indium tin oxide (ITO) coated glass slides within the "O" ring area. The solvent was evaporated at room temperature and the slides were dried overnight under vacuum. Then, ITO slides were assembled in the Vesi Prep Pro and filled with 275 μL of sorbitol solution (1 M). A sinusoidal AC field of 3 V and 5 Hz was applied for 2 h at 25 °C temperature. The GUV solution prepared was collected and stored at 4 °C.

Conductance measurements

Conductance measurements were performed using the Port-a-Patch setup (Nanion, Munich, Germany). First, a borosilicate glass chip (NPC chip, Nanion, Germany) with 3-5 mΩ was loaded with symmetrical working buffer containing 1 M KCl, and 10 mM HEPES (pH 7.0) in both *cis* and *trans* compartments and Ag/AgCl electrodes were placed on both sides of the NPC chip. Next, bilayer membrane with >1 Giga Ohm resistance was constructed across the micrometer-sized aperture in the

NPC chip by adding GUV suspension and applying a small negative pressure (-20 mbar). **MG**/*h-TELO* (60 µM) was added to the *cis*-side of the chip. Current traces were recorded using an HEKA EPC 10 patch clamp amplifier with a built-in 1 kHz 4 pole Bessel low-pass filter and a Digidata 1322A digitizer. The I-V curve was generated using a voltage ramp from -60 mV to +60 mV. For ion selectivity, similar experiments were performed in 10 mM HEPES (pH 7.0) buffer containing 1 M NaCl or having a KCl gradient of 1 M : 0.5 M (*cis*: *trans*). Data analysis was performed using Clampfit 10.6 software.

Circular dichroism (CD) spectroscopy. CD spectra were recorded on a JASCO J-815 spectrophotometer using a 250 µL quartz cuvette of 1-mm optical path length. The CD spectrum of **MG**/*h-TELO* (20 µM) in LUVs suspension (40 µM, 20 µL) was recorded in 10 mM HEPES, 100 mM KCl, pH 7.4 at 25 °C. The suspension was centrifuged at 14000 rpm and the CD spectrum of the supernatant was investigated. Next the vesicles were resuspended in the supernatant followed by the incremental addition of Thiazole orange (TO) (0-4 equiv.) or cytosine (0-3 equiv.). After each addition, the suspension was equilibrated for 2 min, then centrifuged and then the spectrum was recorded. The CD data are a representation of three averaged scans and all CD spectra are baseline corrected for signal contributions from the buffer. The final analysis of the data was carried out by using Origin 8.0.

Measurement of reversal potential. The reversal potentials were measured in bi-ionic conditions. The internal NPC chip solution (*trans* side) used was 150 mM XCl (X = Li^+, Na^+, K^+, Rb^+ and Cs^+) and 10 mM HEPES buffered with Tris (pH 7.4). The external chip solution (*cis* side) contained 150 mM KCl and 10 mM HEPES buffered with Tris (pH 7.4). **MG**/*h-TELO* (60 µM) was added to the *cis* side of the NPC chip. The membrane potential was stepped to +100 mV for 1 s to activate the channels fully and then switched to various testing potentials (-90 mV to 70 mV). The peak tail currents were recorded to generate an I-V curve for the determination of the reversal potential. The sample traces and the I-V curves shown in each figure were obtained from current recordings on the same patch. The values of reversal potential are the mean ± SEM from at least five measurements from different patches. The ion permeability ratios of **MG**/*h-TELO* and its mutants were calculated with the following equation:

$$\frac{P_X}{P_K} = [K]_{int} \exp \frac{\frac{E_{rev}F}{RT}}{[X]_{ext}}, \quad (X = Na^+, \mathrm{K}^+, Li^+, Rb^+, Cs^+) \qquad .... (2)$$

where, E_{rev} is the reverse potential, F is Faraday's constant, R is the gas constant, and T is the absolute temperature. The liquid junction potentials were corrected in our measurements.

Supplementary Figures

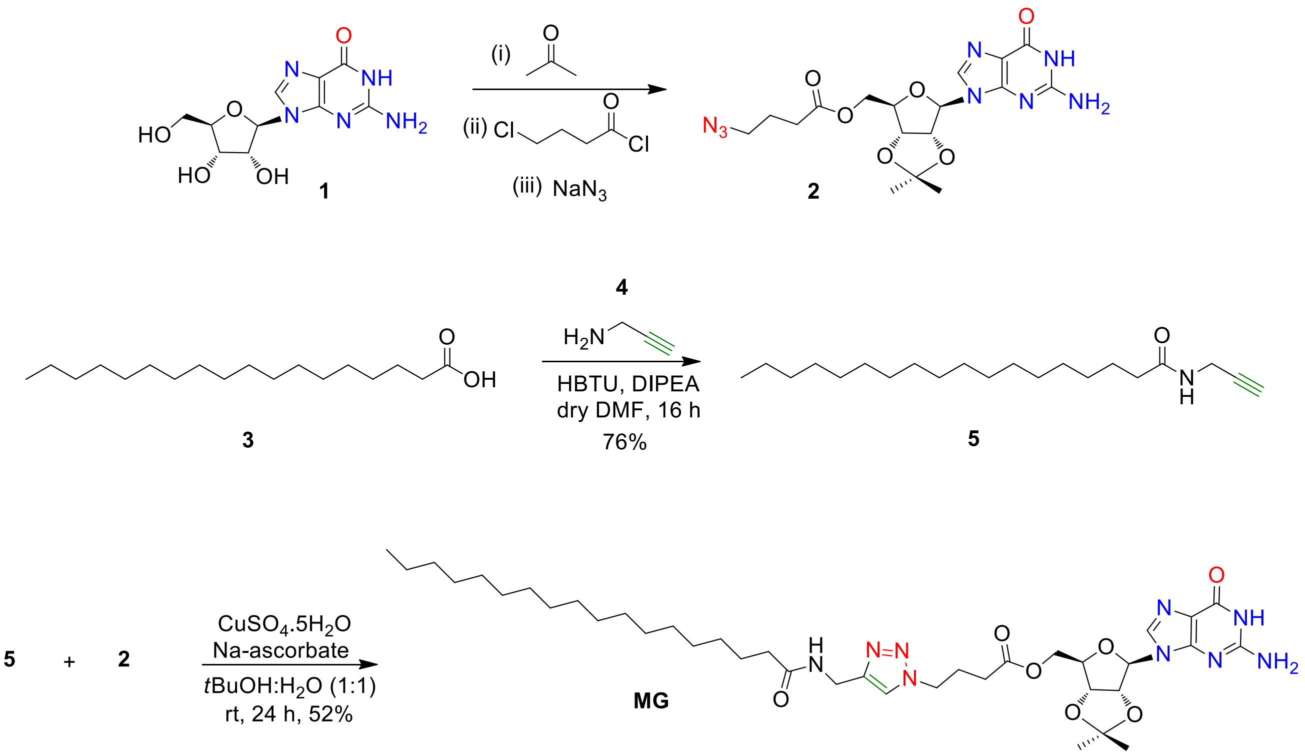

Supplementary Figure 1. Outline of synthesis of **MG.**

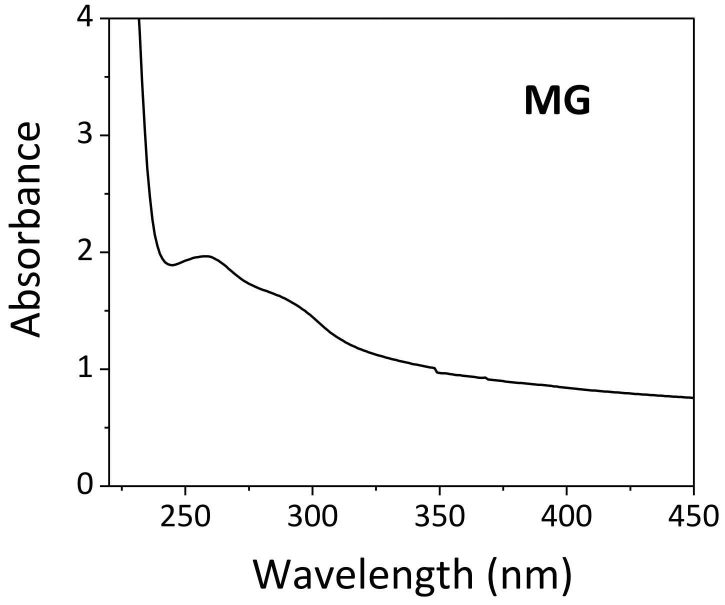

Supplementary Figure 2. UV-Vis spectroscopy. UV-vis spectra of **MG** (10 µM) in 10 mM HEPES, 100 mM KCl buffer (pH 7.4).

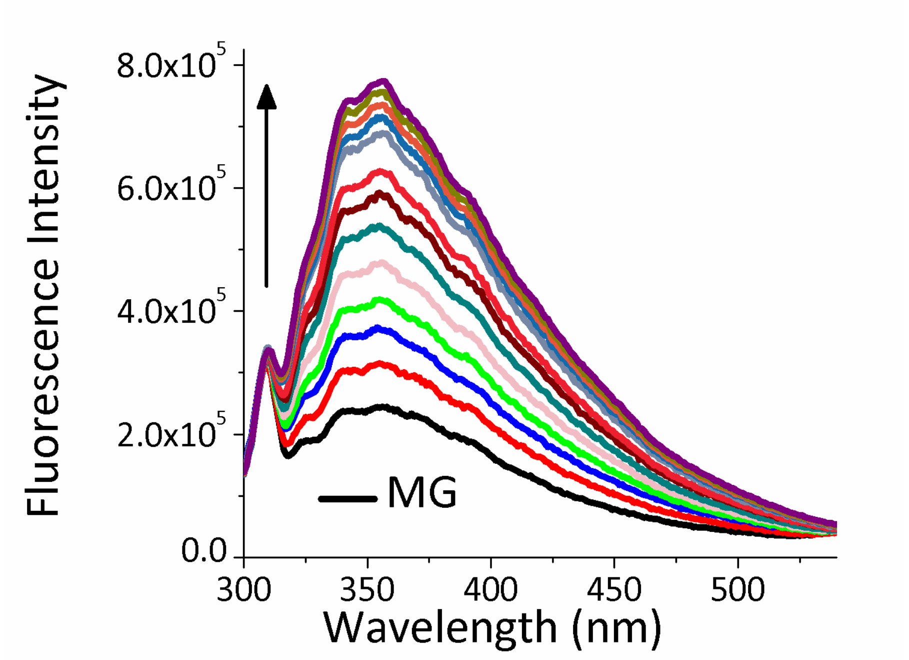

Supplementary Figure 3. Fluorescence binding titration of **MG** with *h-TELO*. Fluorescence spectra of **MG** (10 µM) (excitation wavelength = 265 nm) in 10 mM HEPES, 100 mM KCl buffer (pH 7.4) upon titration with *h-TELO* (0-6 µM).

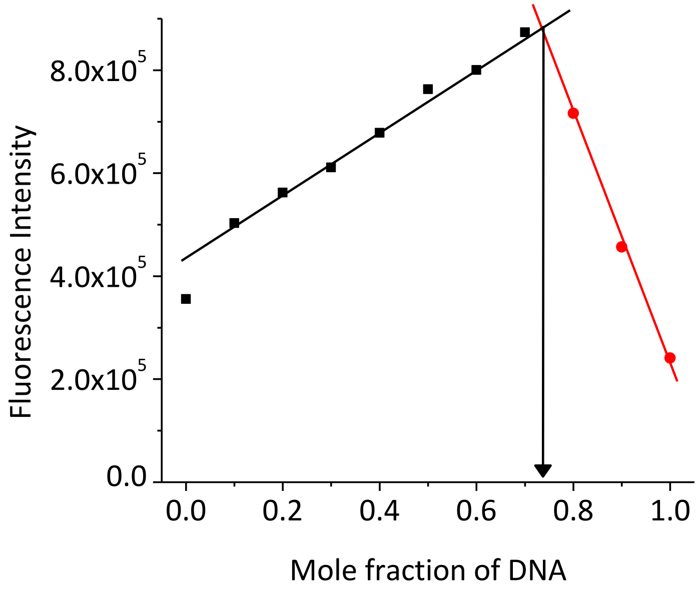

Supplementary Figure 4. Job's plot analysis. Job's plot analysis by continuous variation in the mole fraction of **MG** (0, 0.1, 0.2, 0.3, 0.4, 0.5, 0.6, 0.7, 0.8, 0.9, 1.0) in the presence of *h-TELO* (mole

fraction: 1.0, 0.9, 0.8, 0.7, 0.6, 0.5, 0.4, 0.3, 0.2, 0.1, 0) in 10 mM HEPES, 100 mM KCl buffer (pH 7.4).

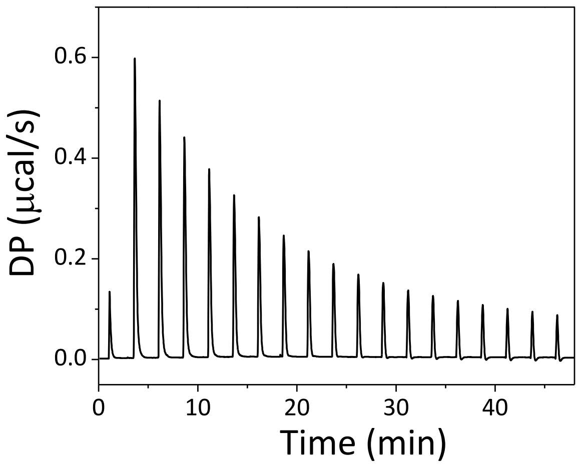

Supplementary Figure 5. Isothermal titration calorimetry (ITC). ITC analysis of the binding of **MG** (100 µM) to *h-TELO* DNA (5 µM). Raw data of titrations, in which the power output in microcalories per second, is measured as a function of time in minutes.

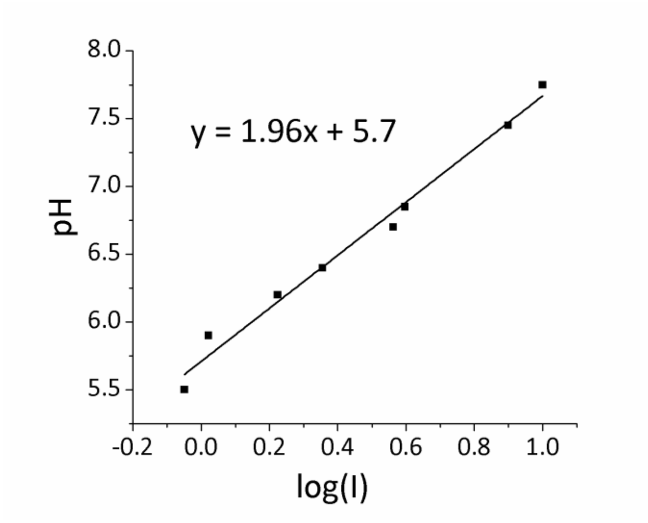

Supplementary Figure 6. Standard curve showing the change in HPTS fluorescence intensity (in log scale) with pH.

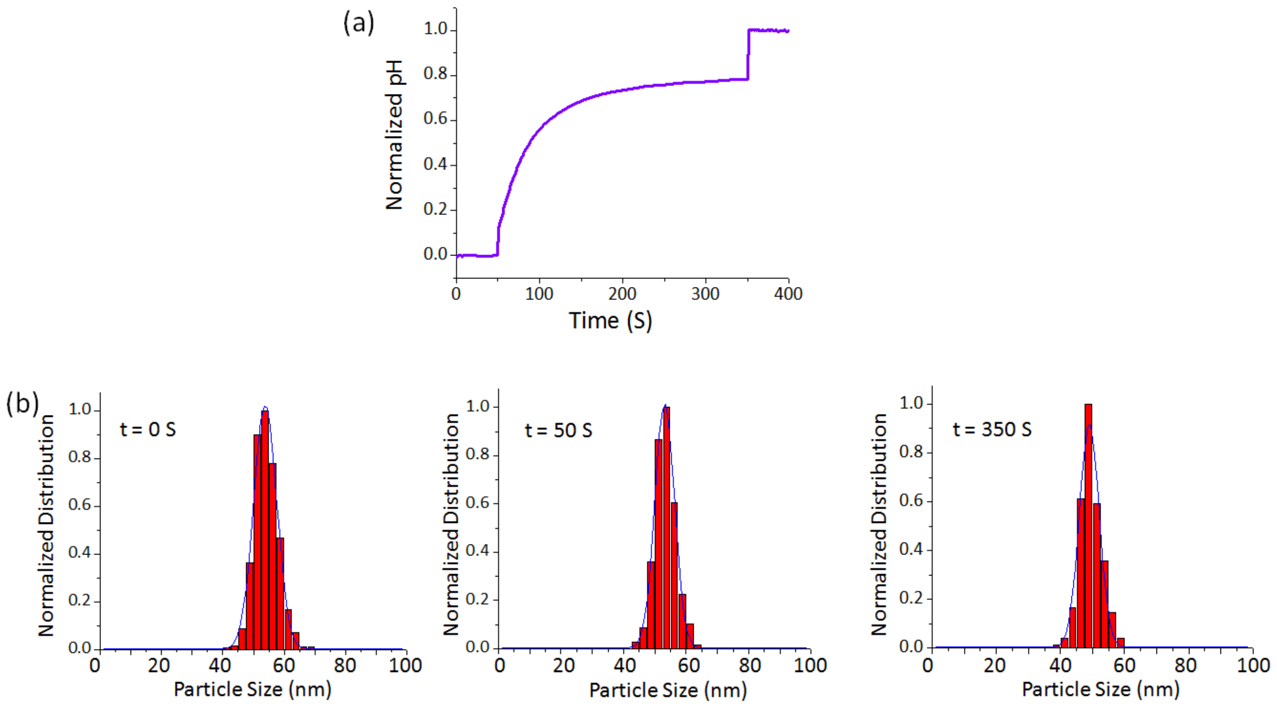

Supplementary Figure 7. Dynamic light scattering (DLS) analysis. Size distribution of 20 µM **MG**/*h-TELO* embedded in 40 µM of LUV suspension in 10 mM HEPES, 100 mM KCl, pH 7.4 at different time points; t = 0 s, 50 s and 350 s.

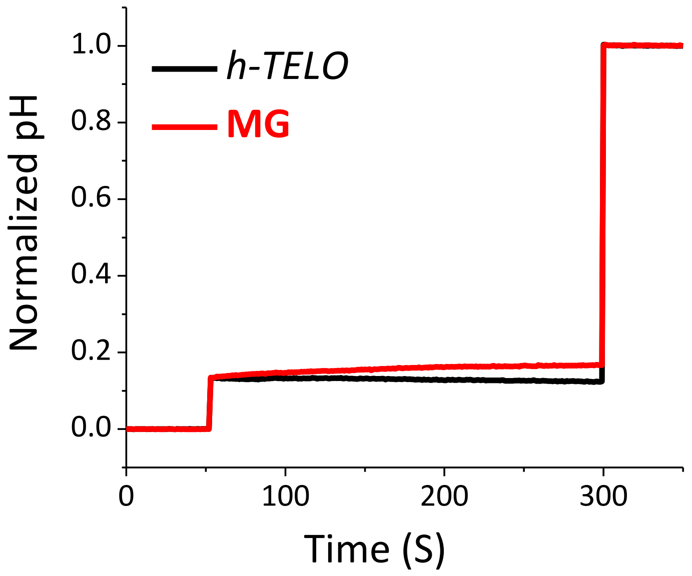

Supplementary Figure 8. Fluorescence based vesicle experiments (Control data). Changes in normalized pH inside LUV as a function of time with increasing addition of either **MG** or *h-TELO* (0-60 µM). Buffer composition, external buffer: 10 mM HEPES, 100 mM KCl, pH 7.4; internal buffer: 10 mM HEPES, 100 mM NaCl, pH 6.4.

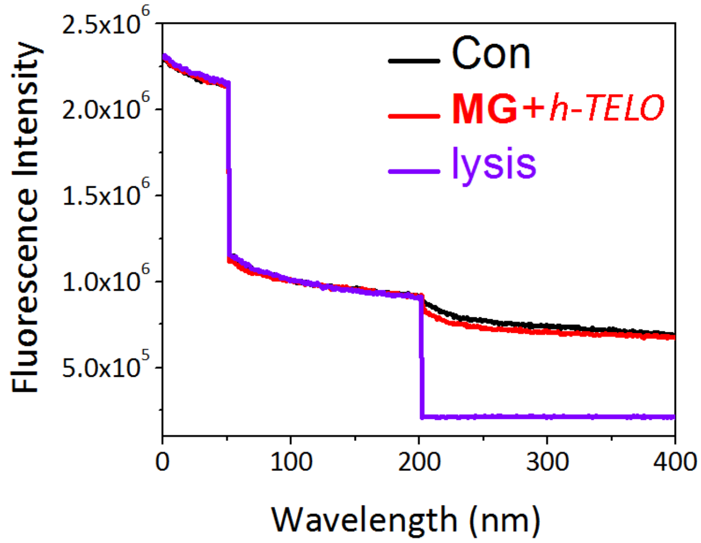

Supplementary Figure 9. Lucigenin assay. Change in fluorescence of Lucigenin entrapped LUVs as a function of time with **MG**/*h-TELO* (60 μM) and after lysis with Triton-X.

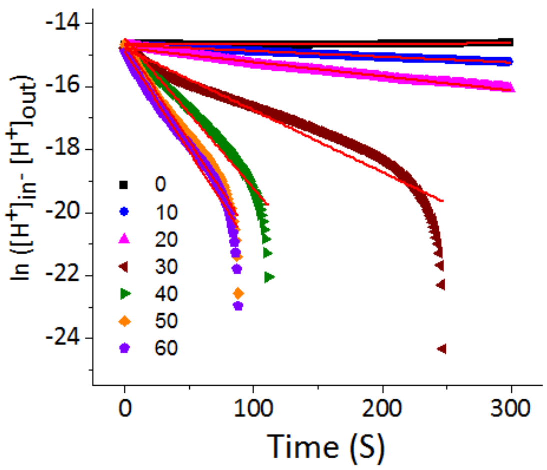

Equation	y = a + b*x		
Adj. R-Square	0.34782	0.99679	0.98634
		Value	Standard Error
B	Intercept	-14.67315	0.00182
B	Slope	1.33656E-4	1.05513E-5
C	Intercept	-14.69558	0.00104
C	Slope	-0.00183	5.9929E-6
D	Intercept	-14.78003	0.00528
D	Slope	-0.00449	3.05604E-5
F	Intercept	-14.78504	0.06124
F	Slope	-0.01967	4.29021E-4
G	Intercept	-14.46162	0.07242
G	Slope	-0.0477	0.00113
H	Intercept	-14.6214	0.07971
H	Slope	-0.0622	0.00156
I	Intercept	-14.87952	0.0813
I	Slope	-0.06345	0.0016

Supplementary Figure 10. Pseudo first order kinetics of transport by **MG**/*h-TELO* (0-60 μM). Linear fitting of ln ($[H^+]_{in}$ - $[H^+]_{out}$) versus time at 0-60 μM of **MG**/*h-TELO* in the bilayer membrane. Buffer composition, external buffer: 10 mM HEPES, 100 mM KCl, pH 7.4; internal buffer: 10 mM HEPES, 100 mM NaCl, pH 6.4.

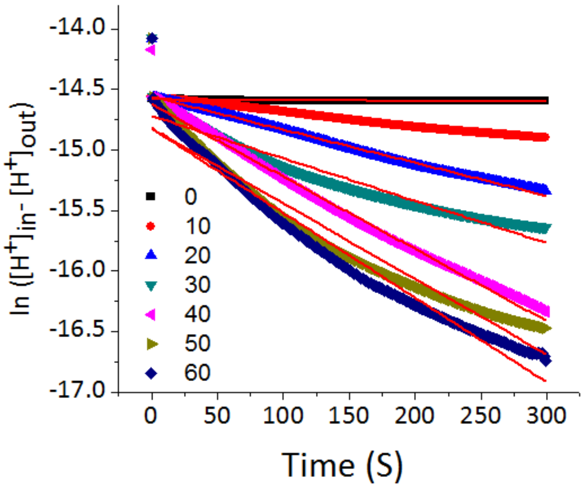

Equation	y = a + b*x		
Adj. R-Square	0.65194	0.99554	0.98271
		Value	Standard Error
B	Intercept	-14.5832	4.46523E-4
B	Slope	-6.12163E-5	2.58446E-6
C	Intercept	-14.56695	7.83087E-4
C	Slope	-0.00117	4.53249E-6
D	Intercept	-14.55926	0.00362
D	Slope	-0.00273	2.09727E-5
E	Intercept	-14.71831	0.00889
E	Slope	-0.00351	5.14526E-5
F	Intercept	-14.62162	0.00548
F	Slope	-0.00598	3.17257E-5
G	Intercept	-14.81758	0.01367
G	Slope	-0.00624	7.91387E-5
H	Intercept	-14.81972	0.01255
H	Slope	-0.00701	7.26221E-5

Supplementary Figure 11. Pseudo first order kinetics of transport by **MG**/*h-TELO* (0-60 µM). Linear fitting of ln ($[H^+]_{in}$ - $[H^+]_{out}$) versus time at 0-60 µM of **MG**/*h-TELO* in the bilayer membrane. Buffer composition, external buffer: 10 mM HEPES, 100 mM NaCl, pH 7.4; internal buffer: 10 mM HEPES, 100 mM KCl, pH 6.4.

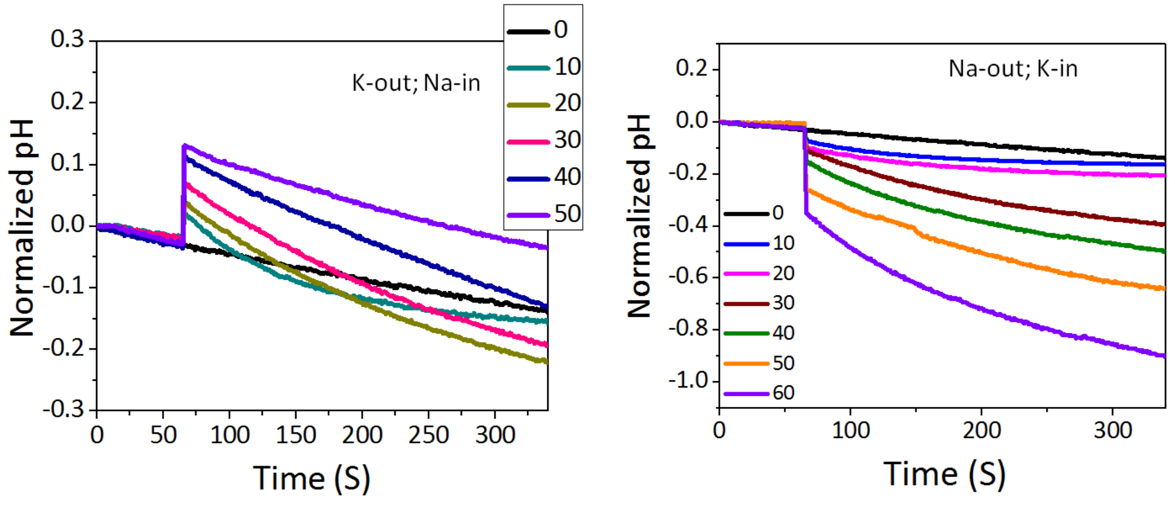

Supplementary Figure 12. Passive transport by **MG**/*h-TELO*. Changes in normalized pH inside LUVs as a function of time with increasing addition of **MG**/*h-TELO* (0-60 μM). The metal ions (100 mM) present in and out of vesicle are indicated in the figure.

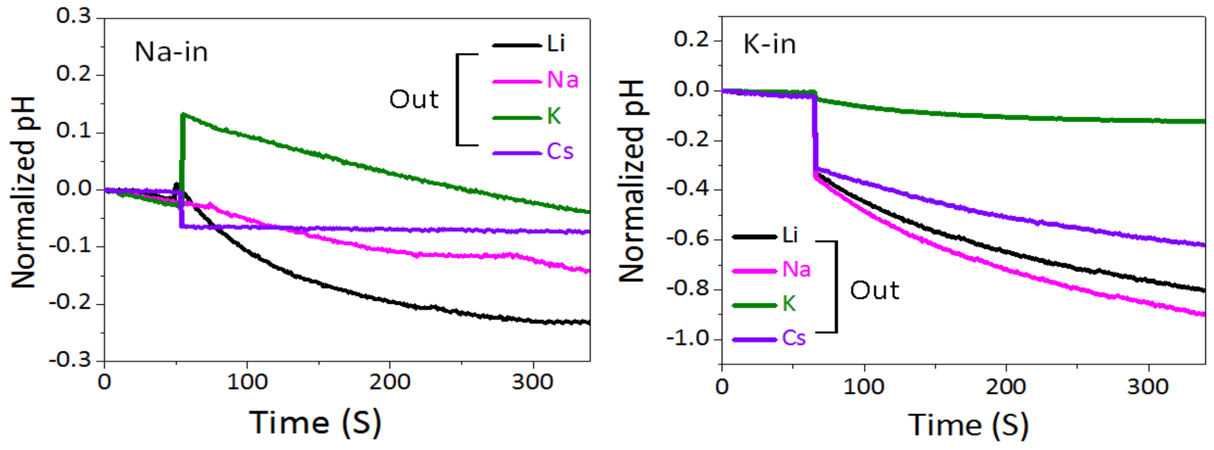

Supplementary Figure 13. Passive transport by **MG**/*h-TELO*. Changes in normalized pH inside LUVs as a function of time in external buffer containing different metal ions with **MG**/*h-TELO* (0-60 μM). The metal ions (100 mM) present in and out of vesicle are indicated in the figure.

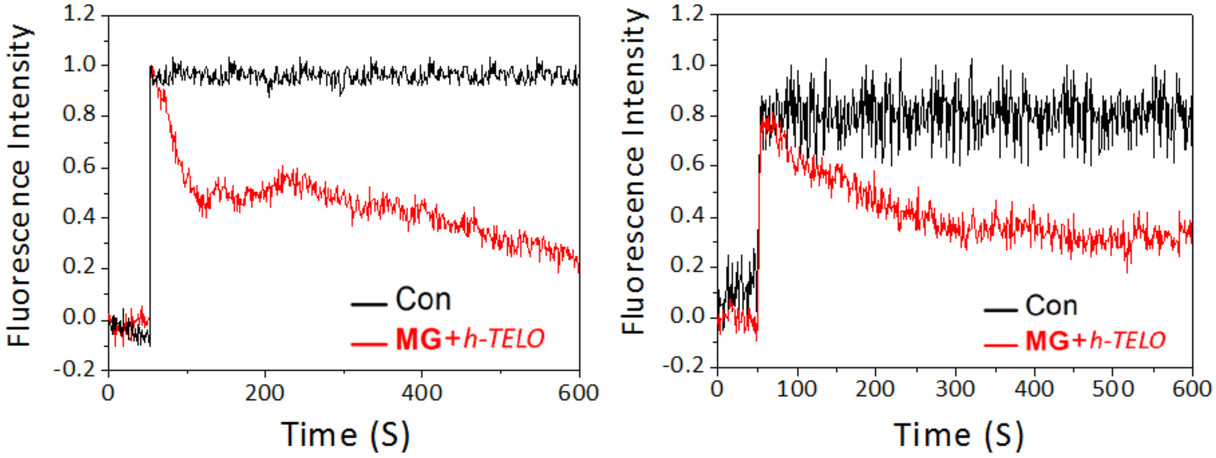

Supplementary Figure 14. Safranin O assay. Change in fluorescence of safranin O entrapped LUV as a function of time with 60 µM **MG**/*h-TELO* (Left) and 30 µM **MG**/*h-TELO* (Right) [Internal buffer: 10 mM HEPES, 100 mM NaCl pH 6.4; External buffer: 10 mM HEPES, 100 mM KCl pH 6.4].

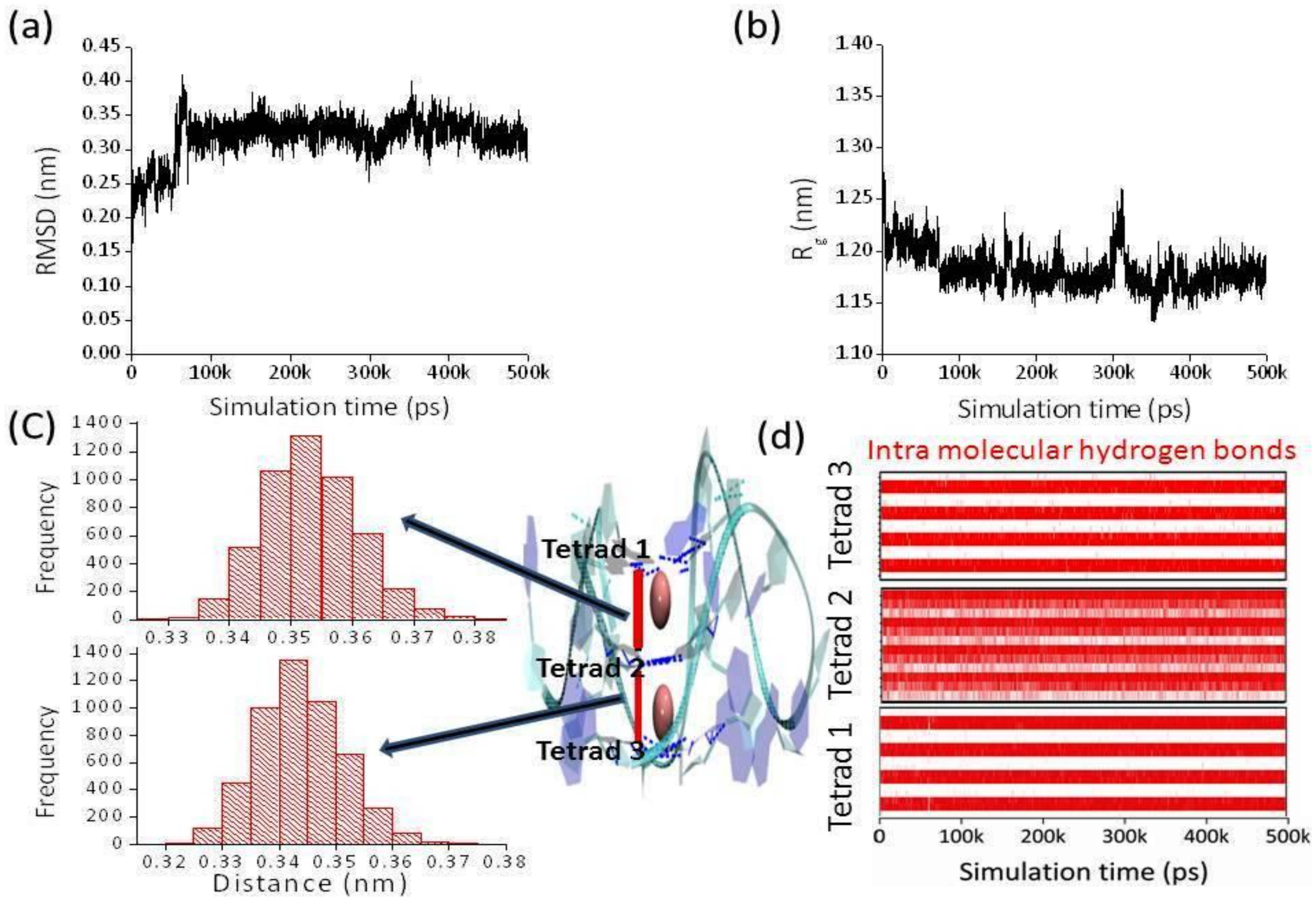

Supplementary Figure 15. Validation of the force field and water model for quadruplex simulation. (a) Variations of root mean square deviation, RMSD, and (b) Radius of gyration, R_g, with simulation time are shown. (c) Histogram representation of the distance distributions obtained from equilibrium molecular dynamics simulation between Quartet 1 and Quartet 2 (upper panel); Quartet 2 and Quartet 3 (lower panel) are shown. (d) Variation of intramolecular hydrogen bonding map for the three quartets of the quadruplex is shown.

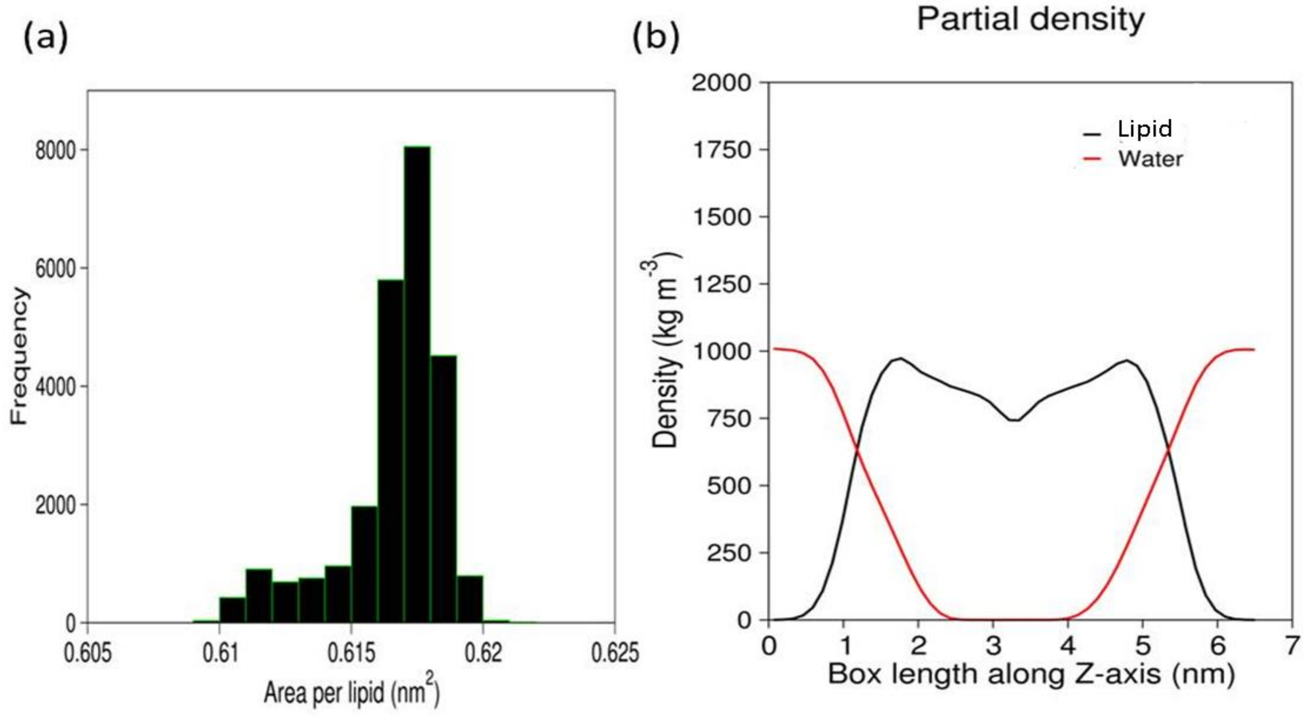

Supplementary Figure 16. Validation of forcefield for lipid simulation. (a) Distribution of area per lipid calculated from 500 ns simulation trajectory. (b) Density profile of lipid and water obtained from equilibrium simulation.

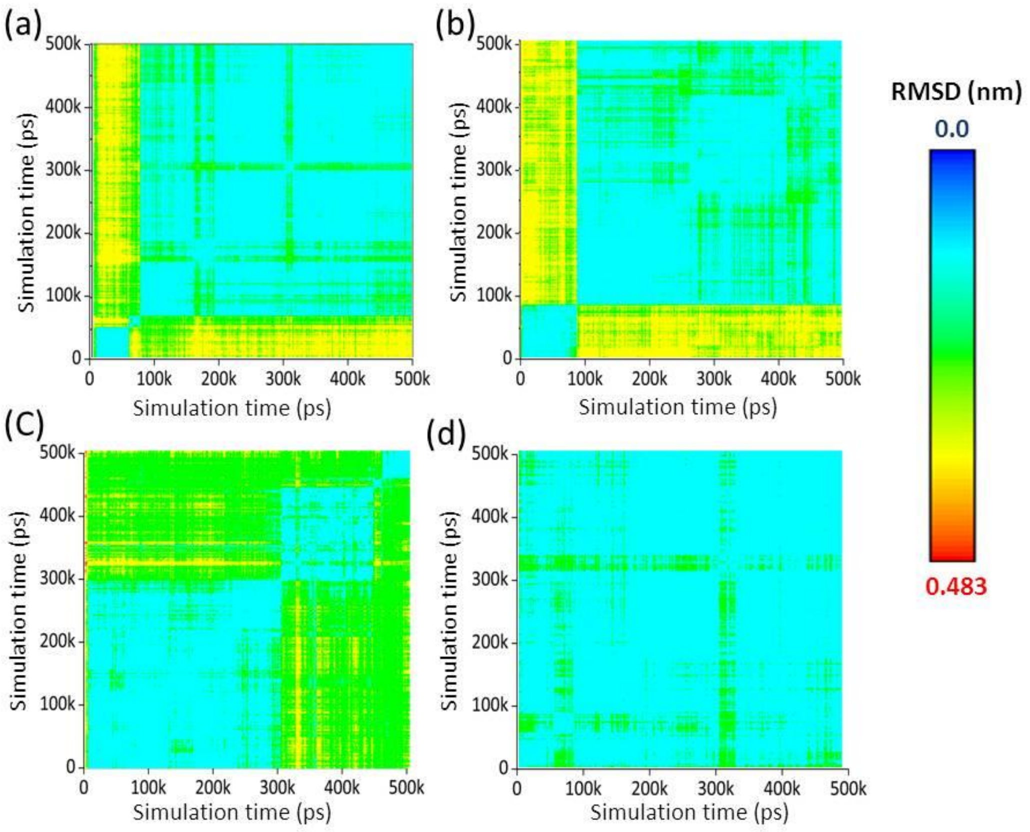

Supplementary Figure 17. RMSD matrix obtained from the 500 ns MD simulation of (a) *h-TELO* and assemblies of (b) **MG**/*h-TELO* (1:1), (c) **MG**/*h-TELO* (2:1) and (d) **MG**/*h-TELO* (3:1).

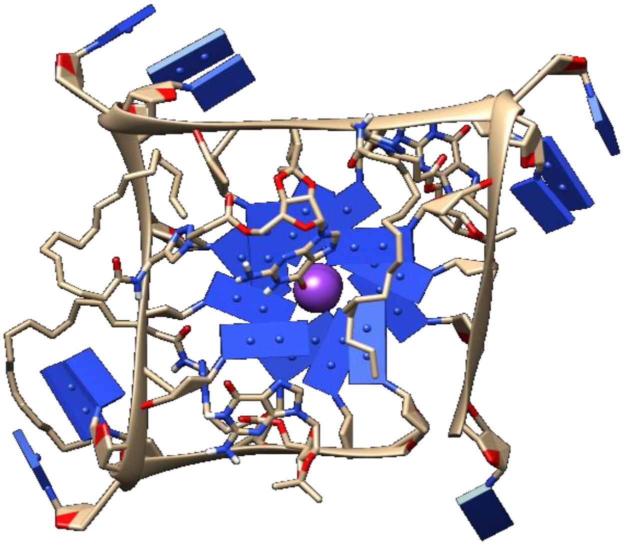

Supplementary Figure 18. Structure of the 3:1 **MG**/*h-TELO* assembly (*h-TELO* PDB: 1KF1) in aqueous solution.

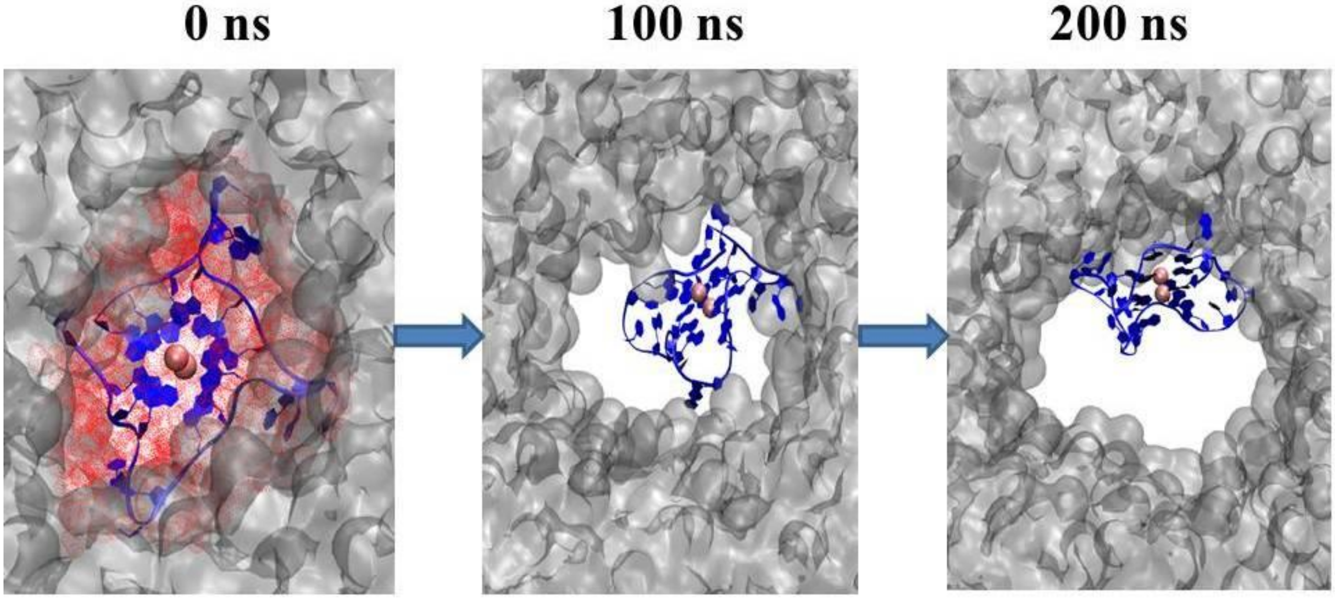

Supplementary Figure 19. Structural evolution of the supramolecular ionophore embedded within the lipid bilayer was simulated without the presence of bound ligand during the 200 ns equilibrium simulation. Quadruplex is rendered in blue ribbon and the K^+ ions residing the central lumen of the quadruplex is rendered in brown CPK mode. Membrane is shown as gray surface. Water of the ion pore is shown as red dots in initial structure only.

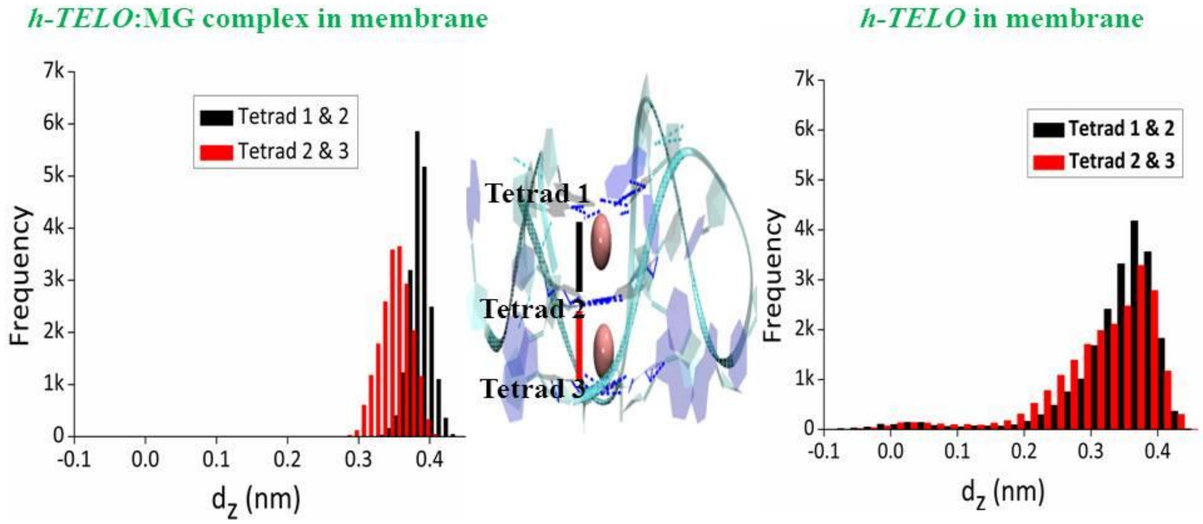

Supplementary Figure 20. Variations of the distance vector along Z-axis between quartets are shown for **MG**/*h-TELO* ionophore embedded in lipid bilayer (left) and **MG** free *h-TELO* (right) within the water pore. The color code (red and black) shown in the schematic representation indicates the distance between quartets 1-2 and quartets 2-3.

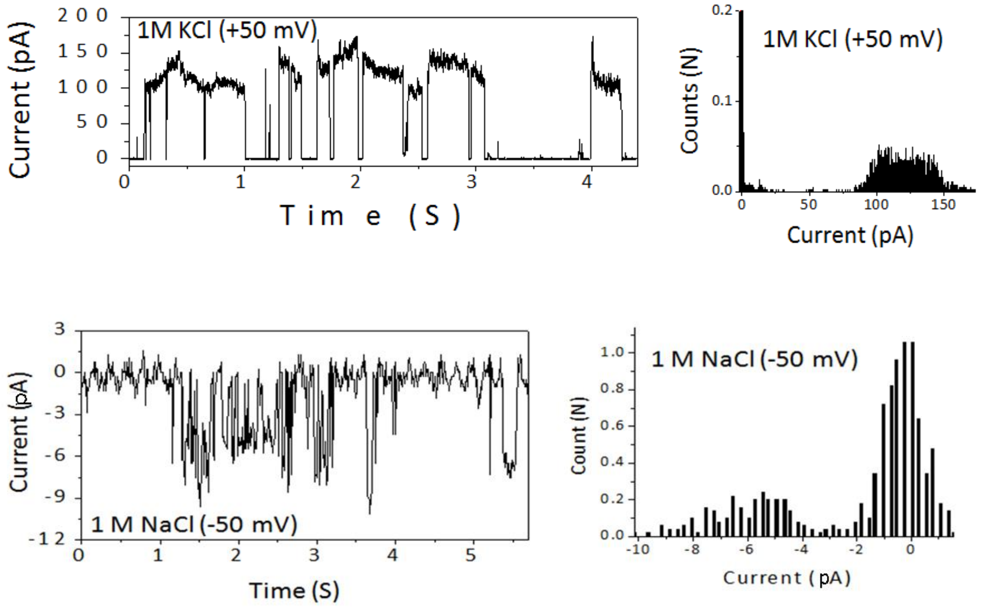

Supplementary Figure 21. Voltage clamp analysis. Single-channel current traces recorded at +80 mV (top) in holding potentials in 1 M symmetrical KCl solution and −50 mV (bottom) in 1 M symmetrical NaCl solution in the presence of **MG**/*h-TELO*. All point histograms generated from the corresponding current traces at +80 mV and −50 mV have been presented on the right.

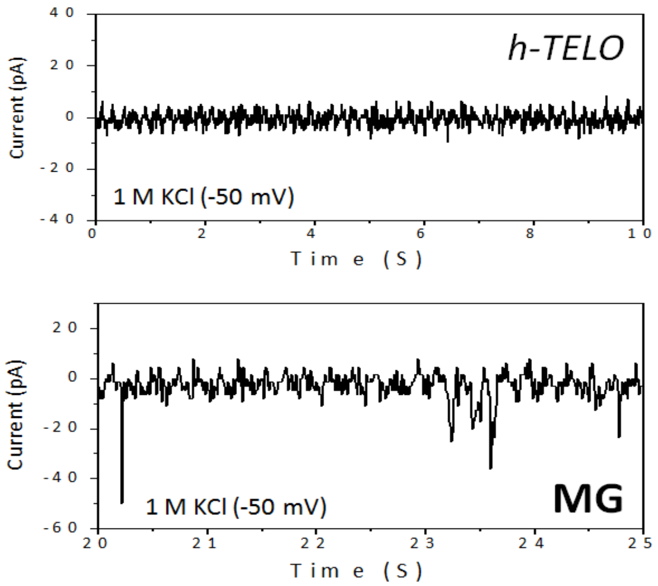

Supplementary Figure 22. Voltage clamp analysis. Single-channel current traces recorded at -50 mV in holding potentials in 1 M symmetrical KCl solution in the presence of *h-TELO* (top) and heat annealed **MG** in K^+ ion (bottom).

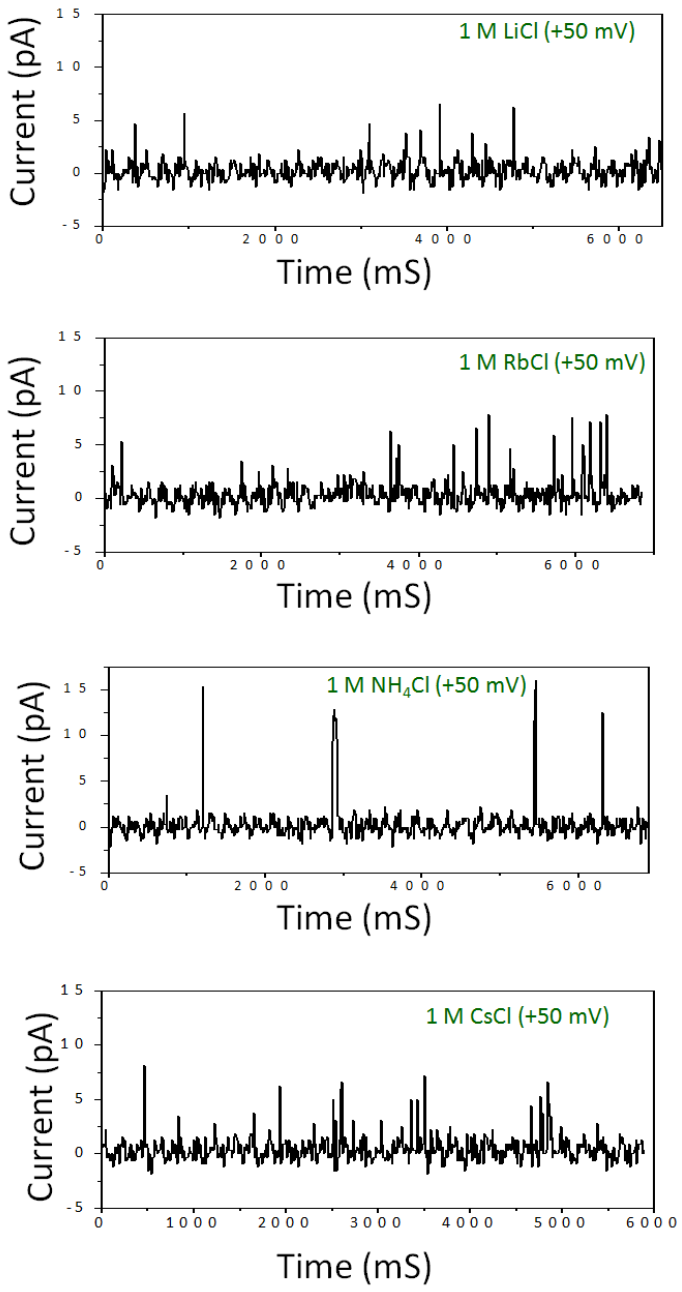

Supplementary Figure 23. Voltage clamp analysis. Single-channel current traces recorded at +50 mV holding potentials in **MG**/*h-TELO* treated vesicles in 1 M symmetrical solution of MCl (M = Li^+, Rb^+, NH_4^+ and Cs^+).

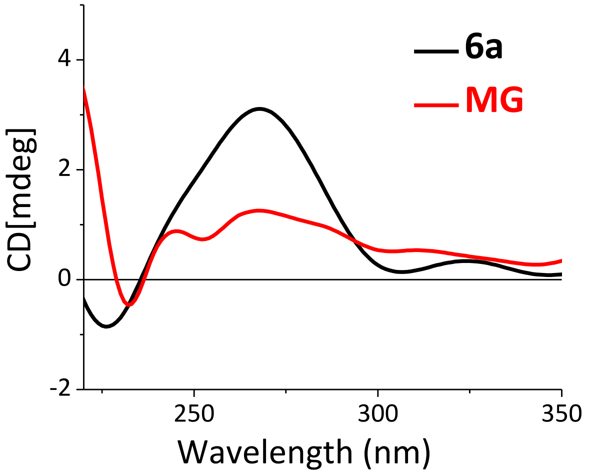

Supplementary Figure 24. CD spectroscopy. CD spectra of heat annealed **MG** and **6a** in 10 mM HEPES, 100 mM KCl, pH 7.4. Compound **6a** has been previously reported.[4]

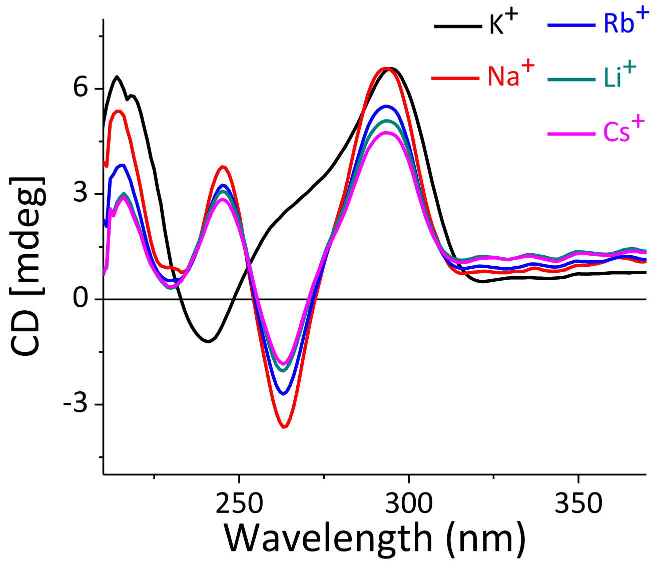

Supplementary Figure 25. CD spectroscopy. CD spectra of **MG**/*h-TELO* in 10 mM HEPES, 100 mM XCl (Li^+, Na^+, K^+, Rb^+ and Cs^+), pH 7.4.

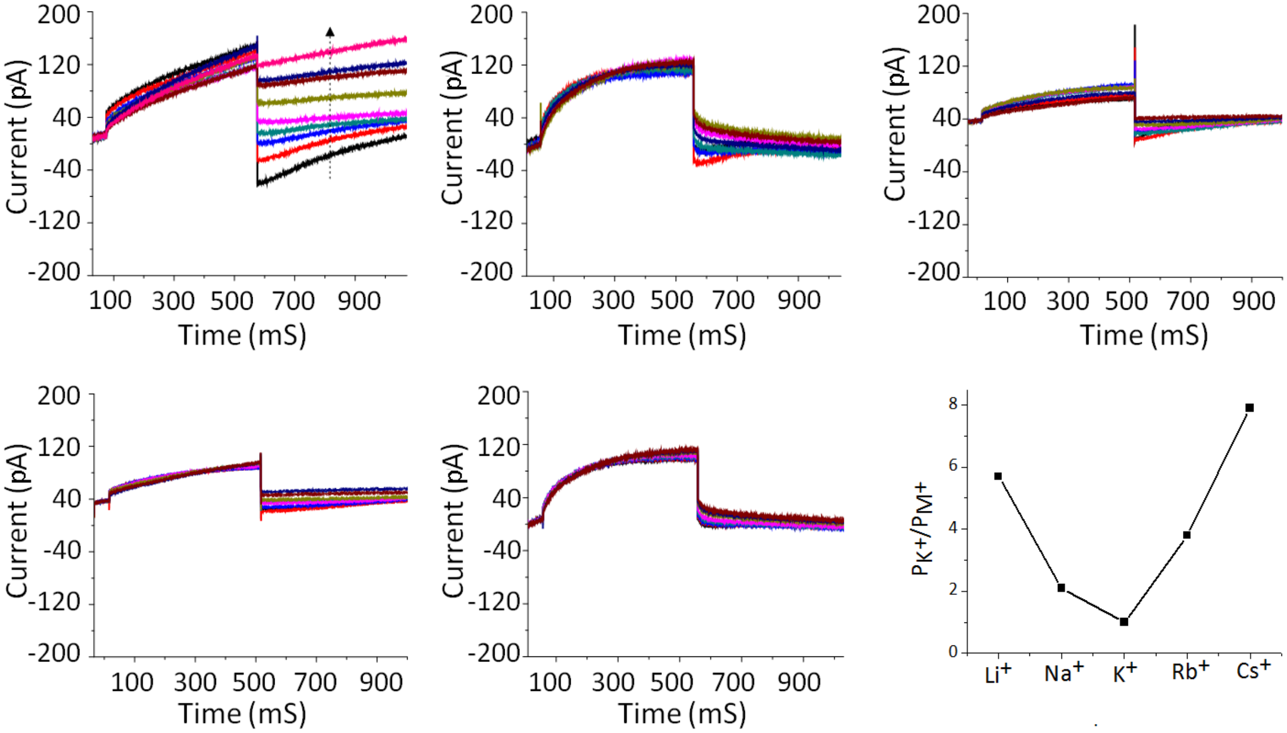

Supplementary Figure 26. Measurement of reversal potential. Sample traces of **MG**/*h-TELO* (60 µM) in vesicles measured in 150 mM KCl and 10 mM HEPES buffered with Tris (pH 7.4) (external solution) and 150 mM XCl (X = Li^+, Na^+, K^+, Rb^+ and Cs^+) and 10 mM HEPES buffered with Tris (pH 7.4) (internal solution).

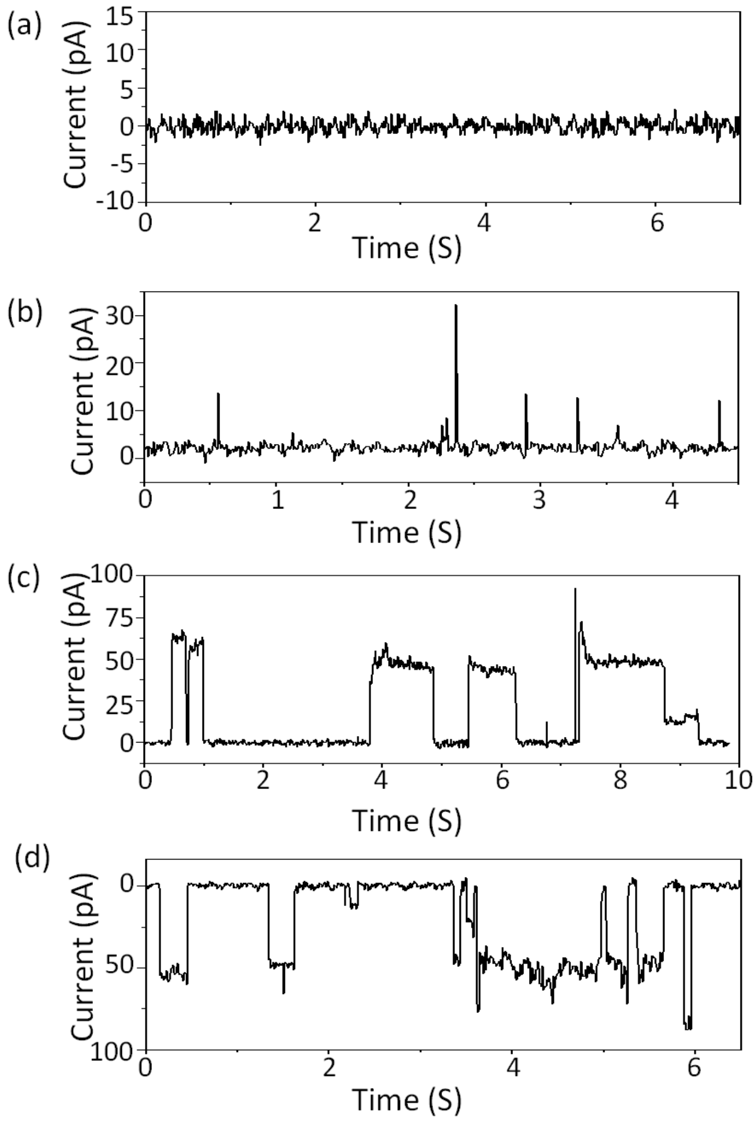

Supplementary Figure 27. Voltage clamp analysis for cell experiments. Single-channel current traces recorded for (a) only CHO cells (+30 mV), (b) only K-562 cells (+30 mV), (c) **MG**/*h-TELO* treated K-562 cells (+30 mV), (d) **MG**/*h-TELO* treated K-562 cells (-30 mV) in 10 mM HEPES, 150 mM KCl, pH 7.4 symmetrical buffer.

Supplementary Notes

Cation transport experiments

The first order initial rate constant was calculated from the slopes of the plot of $\ln([H^+]_{in} - [H^+]_{out})$ versus time, where $[H^+]_{in}$ and $[H^+]_{out}$ are the concentrations of H^+ ions in the intravesicular and extravesicular solutions, respectively.[5] The pH of the extravesicular solution was assumed to be

constant at 7.4 and the corresponding H^+-ion concentration was used for the calculation. The $[H^+]_{in}$values were calculated for each point from HPTS emission intensity using the equation pH = 1.96 × log(I_{460}) + 5.7. The normalized pH has been calculated as follows: pH_{norm} = (pH-$pH_{initial}$)/(pH_{final}-$pH_{initial}$). The $pH_{initial}$ and pH_{final} are the initial (t = 0 s) and final pH (plateau), respectively. The EC_{50} and Hill coefficient n were calculated using the plot of fractional activity Y against the increasing concentrations of **MG**/*h-TELO*. The value of Y was obtained for each curve by normalizing the I_{460} value of the initial (t = 0 s) and final pH (just before lysis) in the 0 to 1 scale. This plot was fitted using Hill equation: $Y=1/(1+ (EC_{50}/[C])^n)$.

Determination of chloride ion selectivity by lucigenin assay

To investigate whether **MG**/*h-TELO* could transport chloride ions (Cl^-) along with alkali cations, lucigenin assay was conducted as follows: LUVs were filled with 225 mM $NaNO_3$ along with the chloride-sensitive dye lucigenin (0.8 mM), and suspended in $NaNO_3$ (225 mM). At t = 50 s, 24 mM NaCl was added followed by the addition of 60 μM **MG**/*h-TELO* at t = 100 s. Subsequent recording of the fluorescence of lucigenin indicated that **MG**/*h-TELO* did not promote influx of Cl^- as evident from the negligible change in lucigenin fluorescence intensity compared to the control (Supplementary Figure 13). In contrast, when Triton-X was used as a positive control, we observed a sharp decrease in fluorescence intensity of lucigenin due to the presence of Cl^- after membrane disruption.

Membrane polarization experiments

To investigate whether the passive transport of ions by **MG**/*h-TELO* induces membrane hyperpolarization, fluorescence assay was performed using potential sensitive dye safranin O (Supplementary Figure 14).[5] Liposomes filled with 100 mM NaCl or KCl in HEPES buffer solution (10 mM, pH 6.4) were suspended in 100 mM NaCl or KCl in HEPES buffer (10 mM, pH 6.4) containing 10 mM safranin O.

Upon addition of **MG**/*h-TELO* (60 μM), a sharp increase in safranin O fluorescence was observed possibly due to the interaction of safranin O with nonpolar sites of **MG**/*h-TELO*. No change in fluorescence behavior was observed when 100 mM NaCl was present in both interior and exterior solutions. Importantly, by carrying out the same experiment with 100 mM KCl instead of NaCl in external buffer, a dose-dependent decrease in fluorescence was observed over time. The change in fluorescence may be attributed to the accumulation of negative charge outside of vesicles due to the

influx of K^+.[5] As expected, the fluorescence intensity of safranin O remained unchanged upon addition of **MG**/*h-TELO* assembly (60 μM) in the absence of K^+ gradient (100 mM KCl was present in both external and internal buffer). These results suggest that **MG**/*h-TELO* can modulate the transmembrane potential in the presence of K^+ gradient.

Molecular modelling studies

All the simulations were performed using GROMACS 2016 molecular dynamics package.[6,7,8] A modified force field particularly developed for DNA simulation, parmbsc1,[9] (http://mmb.irbbarcelona.org/ParmBSC1) with the SPC/E[10] water model was used throughout the study. All the visualizations were carried out using visual molecular dynamics (VMD) software.[11]

Preparation and parameterization of ligand structure

Structure of the ligand, **MG**, was optimised *in vacuo* using AM1 semi-empirical method. Ligand parameters for the parambsc1 were generated using the Antechamber tools[12] where partial charges on each ligand atoms were calculated and assigned based on AM1-BCC charge model. Finally, all the parameters for parambsc1 forcefield compatible with the GROMACS simulation packages were generated using ACPYPE software plugin.[13]

Crystal structure of a 22-mer human telomeric DNA (*h-TELO*) that folds into G-quadruplex was considered for the study.[14] K^+ ions within the central lumen of the G-quadruplex were considered during the simulation. Initially, the suitability of the force field and water model was verified by performing long equilibrium simulation of the G-quadruplex in aqueous solution. G-quadruplex was immersed at the centre of a rectangular box of $57 \times 49 \times 42$ Å^3 dimension containing 7508 SPC/E water molecules. The box dimension was chosen in such a way that the minimum distance between quadruplex atoms and box edges $\geq$ 10 Å. Periodic boundary conditions were used to alleviate the evaporation at the box edges. Appropriate K^+ ions were added to make the system charge neutral. The system was then energy minimised using steepest descent algorithm to reduce the bad contacts in the system. It was followed by 1 ns position restrained simulation in NPT ensemble where the G-quadruplex backbone was restrained with the aid of 1000 kJ $mol^{-1}nm^{-1}$ force in all three direction whereas both water and ions were moving freely. Simulation was performed at 298 K and temperature was maintained during the simulation using the Berendsen thermostat with a time constant for coupling set to 0.1 ps. Pressure was kept constant (1 bar) by employing isotropic Berendsen barostat with the time-constant of 2 ps. Electrostatic interactions were calculated using particle mesh Ewald summation

method with default values for grid spacing and 10 Å distance cut-off was used to cut short-range electrostatic interactions. Finally, 500 ns production simulation was performed in NPT ensemble without any restraining potential on quadruplex using the same parameters and trajectory was saved in every 20 ps. Analysis were carried out using simulation trajectory analysis tools available in GROMACS simulation packages.

Suitability of the force field to study the *h-TELO* quadruplex was examined by running long 500 ns equilibrium simulation and results are shown in the Supplementary Figure 14. Evident from the figure, both the root man square fluctuation, RMSD (Supplementary Figure 15a) and radius of gyration, R_g (Supplementary Figure 15b) remained stable during the simulation timescale which indicate stabilization of the quadruplex structure in its native crystallographic form. The deviation between simulation average structure and the crystal structure was only ~3.2 Å. The *h-TELO* quadruplex structure is characterized with three G-quartets. Each quartet contains four guanine bases aligned in a same plane linked with intramolecular hydrogen bonds. Three G-quartets are stacked on each other. Stacking and intramolecular hydrogen bonds are the two primary driving forces that drive a single DNA strand to fold as quadruplex. Both stacking and intramolecular hydrogen bonds of the G-quartet remained highly stable throughout the simulation timescale (Supplementary Figure 15c & 15d). The same force field with the SPC/E water model was previously shown to perfectly mimic duplex DNA dynamics in μs timescale.[8]

Preparation and validation of the lipid bilayer simulation

All-atom structure of 128 molecule lipid bilayer structure was obtained from the Lipidbook.[15] Slipid force field for this lipid was used successfully with different AMBER force fields (AMBER99SB/AMBER99SB-ILDN/AMBER03) and considered for lipid simulation.[16,17] The force field was then imported to parmbsc1 force field. The suitability of the force field to simulate lipid bilayer was then assessed by running 500 ns equilibrium simulation.

The lipid bilayer contains 64 phospholipids in each layer. The bilayer was then placed at the middle of a simulation box of dimension 63 × 64 × 66 $Å^3$. In X-Y plane, the box dimensions were equal to the dimensions of simulated bilayer. Periodic boundary condition was used. The bilayer was then hydrated with SPC/E water molecules. The system was then minimized by 500 steps using steepest descent algorithm. The optimised system of hydrated lipid-bilayer was then subjected to 500 ns equilibrium run in NPT ensemble at 298 K using the same simulation parameters used for quadruplex simulation.

Suitability of the parambsc1 force field in combination with SPC/E water model for lipid bilayer simulation were studied in terms of area per lipid calculation and density profile of the simulated system (Supplementary Figure 16).

Area per lipid calculated from the simulation is shown in Supplementary Figure 16a. Average area per lipid calculated from the simulation is 0.616 ± 0.002 nm^2. Throughout the simulation timescale the lipid bilayer remain stable without any noticeable distortion which is clearly evident from the density profile of both lipid and water in the simulation box along the Z-axis (Supplementary Figure 16b).

Molecular docking to generate G-quadruplex-ligand assemblies with different stoichiometries

MG/*h-TELO* assemblies with 1:1, 2:1 and 3:1 were constructed using a stepwise docking procedure using AutoDock 4.2.[18] Crystal structure of *h-TELO* was obtained from Protein Data Bank (PDB ID 1KF1).[14] The quadruplex structure was then prepared for docking. Charges on each quadruplex atom was computed using Kollman united-atom charge model and then non-polar hydrogen atoms were merged. Grid maps were created based on an empirical free-energy scoring functions. Grid box was large enough to cover the entire quadruplex. AM1 optimized structure of the ligand, **MG**, prepared in the earlier ligand preparation section was considered as an initial input for docking. The ligand was then prepared for docking by assigning the rotatable bonds and then partial atomic charges were calculated using the Gasteiger–Marsili method. Non-polar hydrogen atoms were then merged. 500 Docking runs were performed. A maximum of 2,500,000 GA operations were carried out on a single population of 150 individuals. Defaults parameter for weights of crossover, mutation and elitism were considered. Lowest energy docked assembly was then considered as a probable 1:1 **MG**/*h-TELO* assembly. This assembly was then considered as an initial input for 2:1 docking. 1:1 assembly was then considered as a rigid receptor and ligand was then docked using the similar procedure. The lowest energy 2:1 docked assembly was then considered as an input and another ligand was then docked on it using similar procedure and the lowest energy docked assembly was then considered as 3:1 **MG**/*h-TELO*.

Molecular dynamics simulation G-quadruplex-ligand assemblies with different stoichiometry

All G-quadruplex-ligand assemblies with different stoichiometries (1:1, 2:1, 3:1) were then subjected to molecular dynamics simulation using AMBER parmbsc1 force field. Each assembly was initially minimized *in vacuo* using steepest descent algorithm and each optimised assembly was then immersed into a cubic box such that the minimum distance between the macromolecules and box edges were ≥ 10

Å. Each assembly was then solvated with SPC/E water and made charge neutral by adding appropriate number of K^+ ions. All the systems were considered to be periodic in all three dimensions. All the systems were again energy minimized in water using steepest descent algorithm, followed by 1 ns position restrained simulation in NPT emsemble where the quadruplex backbone was restrained by applying an external force. Finally 500 ns of production simulations were performed for each system in NPT ensemble at 298 K by employing Berendsen thermostat and pressure was kept constant by coupling with Berendsen barostat. Electrostatic interactions were computed using Particle Mesh Ewald (PME) summation method. Details of the simulation parameters were similar to the parameter used to simulate the quadruplex.

Root mean square deviation (RMSD) matrix computed for the quadruplex and three different quadruplex-ligand assemblies with different stoichiometries (1:1, 2:1, 3:1) along the simulation trajectories are shown in Supplementary Figure 17. In case of quadruplex simulation, the crystal structure is stable upto 50 ns after that assembly undergoes little structural reorientation and remain stable in remaining simulation timescale. The structure deviates only ~ 3 Å from the crystal structure. Closer look into the trajectory reveals that the three G-quartet remains highly stable, only loop regions that connect the quartet are little flexible during simulation. With progressive binding of the ligand, **MG**, the native crystal structure becomes more and more stable. The 3:1 assembly is highly stable and during the full 500 ns simulation timescale, the native crystal structure remains intact.

Construction of supramolecular ionophore

The equilibrated structure of the lipid bilayer constituted by 128 phospholipids obtained after the 500 ns equilibrium simulation performed in NPT ensemble at 298 K was used to construct large lipid bilayer. The equilibrated bilayer was then replicated in X-Y plane 4 times to generate bilayer of 512 phospholipids. The bilayer was then placed at the middle of a simulation box with dimension of 124 × 126 × 100 Å^3. In X-Y plane box dimension was equal to the dimension of the bilayer. Periodic boundary condition was used. The bilayer was then hydrated on both side of the bilayer with SPC/E water molecules. The system was then minimized using steepest descent algorithm. The optimised system of hydrated lipid-bilayer was then subjected to 200 ns equilibrium run in NPT ensemble at 298 K. Same simulation parameters used to simulate the relatively small lipid bilayer were used to simulate this large lipid bilayer. An average structure of the large lipid bilayer was obtained from the last 100 ns of the simulation trajectory and considered further to construct the ionophore.

The average structure of the 3:1 **MG**/*h-TELO* assembly was obtained from the last 400 ns of the equilibrium simulation. This structure was then inserted within the large hydrated lipid bilayer in such a way that K^+ filled pore of the *h-TELO* were aligned along the width of the membrane in Z-axis. System was then made charge neutral by adding appropriate number of K^+ and Cl^- ions. Then the system was subjected to 1 ns membrane insertion simulation using the membed protocol. The simulation was carried out at 298 K using the same thermostat and barostat with similar parameters, mentioned earlier. During the simulation, those lipid molecules come in close contact with the inserted G-quadruplex-ligand assembly were removed. Finally, when all the steric classes were removed then the system was equilibrated during the remaining simulation time. Final system contains 3:1 **MG**/*h-TELO* assembly embedded in phopholipid bilayer contains 488 lipid molecules and 31600 solvent molecules. The system was then made charge neutral by adding appropriate number of K^+ and Cl^- ions such that the salt concentration of the system became 200 mM. Periodic boundary condition was used to simulate ionophore. The system was then energy minimized using steepest descent algorithm. The system was then subjected to 100 ns position restrained simulation in NPT ensemble at 298 K where the G-quadruplex backbone was restrained only whereas all the lipids, water molecules and ions are free to move. Temperature was kept constant by coupling with an external thermostat using the Berendsen algorithm with a coupling constant of 0.1 ps. Pressure was kept constant (1 bar) by employing isotropic Berendsen barostat with the time-constant set to 2 ps. Electrostatic interactions were calculated using particle mesh Ewald summation method with default values for grid spacing. Finally, 200 ns production run was performed in NPT ensemble and trajectory was saved in every 20 ps.

In a control simulation, the supramolecular ionophore embedded within the lipid bilayer was simulated without the presence of bound ligand using the same protocol used to simulate the ionophore (discussed above) and results are shown in Supplementary Figure 18.

Molecular dynamics simulations for ionic current calculation

To measure the ionic current conducted by the ionophore computationally, simulations were carried out in NP_zAT ensemble in the presence of an external applied electric field. A voltage drop across the system was generated by applying an external electric field along the Z-axis. We simulated the system at +100 mV, +75 mV, +50 mV, +25 mV and -25 mV, -50 mV, -75 mV, -100 mV. Each simulation was repeated three times at 298 K. Nosé-Hoover thermostat was used maintain temperature of the simulation at 298 K with a time constant of 0.2 ps for coupling. Area of the X-Y plane remains constant throughout the simulation timescale. Semi-isotropic pressure coupling was applied along the Z-axis

using Berendsen barostat with a time constant for coupling set to 5 ps and a compressibility of 4.5×10^{-5} bar^{-1} along the Z-axis. External electric field with appropriate strength was applied along the Z-axis such that the specific voltage drop was attained. Each Simulation was carried out ~ 80 ns where last 50 ns was used to calculate the current conducted by the system to make sure that each system attained the stationary state.

We used the total intensity method[19,20] to compute the instantaneous current conducted by each system in the presence of a voltage gradient across the system. This method computes total current flow across the system. It is noteworthy that in the stationary state, the total current flow in the system essentially equals to the current through the ionophore. Instantaneous current flow is computed using the following equation

$$I(t) = \frac{1}{\Delta t L} \sum_{i=1}^{N} q_i [z_i(t + \Delta t) - z_i(t)] \ldots. (3)$$

Where, z_i denotes Z-coordinate of an ion at particular time and q_i is the charge of the particular ion. L is the simulation box length along Z-axis and Δt is the time interval. We used 10 ps time interval as was used in the original work. Total current flows during a time period was calculated by integrating the instantaneous current. The average current was then computed by linear fitting of the cumulative current. Current conducted by K^+ and Cl^- was also calculated using the same equation.

As evident from Supplementary Figure 19, in the absence of bound ligand, quadruplex diffused away from the ionophore pore and its structure was also distorted. As the quadruplex diffuses to the membrane surface where it interacted with the phosphate group of the lipid bilayer non-specifically and then starts to unfold. During the simulation timescale, we calculated the stability of the three G-quartets by measuring the distance vector along the Z-axis between different quartets and the results are shown in Supplementary Figure 20.

As evident from Supplementary Figure 20, when the ligand **MG** bound with the quadruplex in the ionophore, the quadruplex is highly stable, the stacking was maintained throughout the simulation. However, in the absence of the bound ligand, the quadruplex became unstable and its stacking was completely lost. Distance vector along the z-axis deviated largely during the simulation timescale, indicating unfolding of the quadruplex in the absence of bound ligand within the simulation timescale.

Supplementary references

(1) Pavan Kumar, Y.; Saha, P.; Saha, D.; Bessi, I.; Schwalbe, H.; Chowdhury, S.; Dash, J., Fluorescent Dansyl-Guanosine Conjugates that Bind *c-MYC* Promoter G-Quadruplex and Downregulate *c-MYC* Expression. *ChemBioChem* **2016,** *17* (5), 388-393.

(2) Lohman, T.M. & Mascotti, D.P. Methods in enzymology, Vol. 212 424-458 (Elsevier, 1992).

(3) Job, P. Formation and stability of inorganic complexes in solution. (1928).

(4) Kumar, Y. P.; Das, R. N.; Kumar, S.; Schütte, O. M.; Steinem, C.; Dash, J. *Chem. Eur. J.* **2014**, *20*, 3023.

(5) Sidorov, V.; Kotch, F. W.; Kuebler, J. L.; Lam, Y.-F.; Davis, J. T., Chloride transport across lipid bilayers and transmembrane potential induction by an oligophenoxyacetamide. *J. Am. Chem. Soc.* **2003,** *125* (10), 2840-2841.

(6) Van Der Spoel, D.; Lindahl, E.; Hess, B.; Groenhof, G.; Mark, A. E.; Berendsen, H. J., GROMACS: fast, flexible, and free. *J. Comput. Chem.* **2005,** *26* (16), 1701-1718.

(7) Pronk, S.; Páll, S.; Schulz, R.; Larsson, P.; Bjelkmar, P.; Apostolov, R.; Shirts, M. R.; Smith, J. C.; Kasson, P. M.; Van Der Spoel, D., GROMACS 4.5: a high-throughput and highly parallel open source molecular simulation toolkit. *Bioinformatics* **2013,** *29* (7), 845-854.

(8) Berendsen, H. J.; van der Spoel, D.; van Drunen, R., GROMACS: a message-passing parallel molecular dynamics implementation. *Comput. Phys. Commun*. **1995,** *91* (1-3), 43-56.

(9) Ivani, I.; Dans, P. D.; Noy, A.; Pérez, A.; Faustino, I.; Hospital, A.; Walther, J.; Andrio, P.; Goñi, R.; Balaceanu, A., Parmbsc1: a refined force field for DNA simulations. *Nature methods* **2016,** *13* (1), 55.

(10) Berendsen, H.; Grigera, J.; Straatsma, T., The missing term in effective pair potentials. *J. Phys. Chem*. **1987,** *91* (24), 6269-6271.

(11) Humphrey, W.; Dalke, A.; Schulten, K., VMD: visual molecular dynamics. *J. Mol. Graph.* **1996,** *14* (1), 33-38.

(12) Wang, J.; Wang, W.; Kollman, P. A.; Case, D. A., Automatic atom type and bond type perception in molecular mechanical calculations. *J. Mol. Graph. Model.* **2006,** *25* (2), 247-260.

(13) da Silva, A. W. S.; Vranken, W. F., ACPYPE-Antechamber python parser interface. *BMC Res. Notes* **2012,** *5* (1), 367.

(14) Parkinson, G. N.; Lee, M. P.; Neidle, S., Crystal structure of parallel quadruplexes from human telomeric DNA. *Nature* **2002,** *417* (6891), 876.

(15) Domański, J.; Stansfeld, P. J.; Sansom, M. S.; Beckstein, O., Lipidbook: a public repository for force-field parameters used in membrane simulations. *J. Membrane Biol.* **2010,** *236* (3), 255-258.
(16) Jämbeck, J. P.; Lyubartsev, A. P., Derivation and systematic validation of a refined all-atom force field for phosphatidylcholine lipids. *J. Phys. Chem. B* **2012,** *116* (10), 3164-3179.
(17) Shinoda, W., Mikami, M., Baba, T. & Hato, M. Molecular Dynamics Study on the Effect of Chain Branching on the Physical Properties of Lipid Bilayers: Structural Stability. *J. Phys. Chem. B* **107**, 14030-14035 (2003).
(18) Morris, G. M.; Huey, R.; Lindstrom, W.; Sanner, M. F.; Belew, R. K.; Goodsell, D. S.; Olson, A. J., AutoDock4 and AutoDockTools4: Automated docking with selective receptor flexibility. *J. Comput. Chem.* **2009,** *30* (16), 2785-2791.
(19) Faraudo, J.; Calero, C.; Aguilella-Arzo, M., Ionic partition and transport in multi-ionic channels: a molecular dynamics simulation study of the OmpF bacterial porin. *Biophys. J.* **2010,** *99* (7), 2107-2115.
(20) Aksimentiev, A.; Schulten, K., Imaging α-hemolysin with molecular dynamics: ionic conductance, osmotic permeability, and the electrostatic potential map. *Biophys. J.* **2005,** *88* (6), 3745-3761.
