# Supplementary material for: Ionophore constructed from non-covalent assembly of a G-quadruplex and liponucleoside transports K+-ion across biological membranes
Source: Nat Commun. 2020 Jan 24;11:469. doi: 10.1038/s41467-019-13834-7 (PMC6981123; doi:10.1038/s41467-019-13834-7)
Supplement: Supplementary file 4 — Description of Additional Supplementary Files [file 41467_2019_13834_MOESM4_ESM.pdf]

Title: Supplementary Movie 1

Description: Simulation video showing K^+-ion (blue) transport through the water filled pore (channel, shown in grey) formed by small molecule/G-quadruplex assembly. The two potassium ions (red) constituting G-quadruplex structure do not participate in the transport process but plays an important role in maintaining the integrity of the four stranded structure. The crystal structure of human telomeric G-quadruplex (PDB: 1KF1) has been used in this study. For simplicity, the lipid molecules have been omitted.
